# Time-resolved cryo-EM (TR-EM) analysis of substrate polyubiquitination by the RING E3 anaphase-promoting complex/cyclosome (APC/C)

**DOI:** 10.1038/s41594-023-01105-5

**Published:** 2023-09-21

**Authors:** Tatyana Bodrug, Kaeli A. Welsh, Derek L. Bolhuis, Ethan Paulаkonis, Raquel C. Martinez-Chacin, Bei Liu, Nicholas Pinkin, Thomas Bonacci, Liying Cui, Pengning Xu, Olivia Roscow, Sascha Josef Amann, Irina Grishkovskaya, Michael J. Emanuele, Joseph S. Harrison, Joshua P. Steimel, Klaus M. Hahn, Wei Zhang, Ellen D. Zhong, David Haselbach, Nicholas G. Brown

**Affiliations:** 1 Department of Biochemistry and Biophysics and Lineberger Comprehensive Cancer Center, University of North Carolina, Chapel Hill, NC USA; 2 Department of Pharmacology and Lineberger Comprehensive Cancer Center, University of North Carolina, Chapel Hill, NC USA; 3 Department of Molecular and Cellular Biology, College of Biological Science, University of Guelph, Guelph, Ontario Canada; 4 Research Institute of Molecular Pathology, Vienna BioCenter, Vienna, Austria; 5 Department of Chemistry, University of the Pacific, Stockton, CA USA; 6 School of Engineering, California Polytechnic State University Humboldt, Arcata, CA USA; 7 Department of Computer Science, Princeton University, Princeton, NJ USA; 8 Present Address: College of Future Technology, National Biomedical Imaging Center, Peking University, Beijing, China

**Keywords:** Structural biology, Molecular biology, Mitosis, Ubiquitylation

## Abstract

Substrate polyubiquitination drives a myriad of cellular processes, including the cell cycle, apoptosis and immune responses. Polyubiquitination is highly dynamic, and obtaining mechanistic insight has thus far required artificially trapped structures to stabilize specific steps along the enzymatic process. So far, how any ubiquitin ligase builds a proteasomal degradation signal, which is canonically regarded as four or more ubiquitins, remains unclear. Here we present time-resolved cryogenic electron microscopy studies of the 1.2 MDa E3 ubiquitin ligase, known as the anaphase-promoting complex/cyclosome (APC/C), and its E2 co-enzymes (UBE2C/UBCH10 and UBE2S) during substrate polyubiquitination. Using cryoDRGN (Deep Reconstructing Generative Networks), a neural network-based approach, we reconstruct the conformational changes undergone by the human APC/C during polyubiquitination, directly visualize an active E3–E2 pair modifying its substrate, and identify unexpected interactions between multiple ubiquitins with parts of the APC/C machinery, including its coactivator CDH1. Together, we demonstrate how modification of substrates with nascent ubiquitin chains helps to potentiate processive substrate polyubiquitination, allowing us to model how a ubiquitin ligase builds a proteasomal degradation signal.

## Abstract (web summary)

Here, using cryogenic electron microscopy and cryoDRGN, the authors delineate how the anaphase-promoting complex/cyclosome is reconfigurated to interact with its cognate E2s and thus polyubiquitinate its target. Unexpectedly, multiple ubiquitin moieties are shown to interact with the anaphase-promoting complex/cyclosome machinery, including its activator Cdh1.

## Main

The processivity of molecular machines is paramount to the countless biological processes that enable life. Molecular machines, such as polymerases, ribosomes or proteasomes, commonly use nucleotide-directed motors to potentiate key steps in processive reactions, for example, translocation of substrates^1,2^. Recent advances in cryogenic electron microscopy (cryo-EM) have enabled the description of the physical properties of these machines^3,4^. Thus far, conformations seem to be Boltzmann distributed, without large thermodynamic barriers, and molecules can sample the full space, implying microscopic reversibility at every conformational step^5^. Directionality comes from kinetic asymmetries in processes that stabilize specific conformational states, for example, nucleotide hydrolysis^6^. The fundamentally important ubiquitin (Ub) ligases do not directly bind or hydrolyze nucleotides as a part of their mechanism but still maintain forward-driven, processive activity^7–12^. The complete conformational landscape for any of the >600 Ub ligases during polyubiquitination is unknown^13^. Our current understanding of how these enzymes maintain processivity and ensure that ubiquitination proceeds in a directional manner is therefore incomplete.

**Fig. 1:**
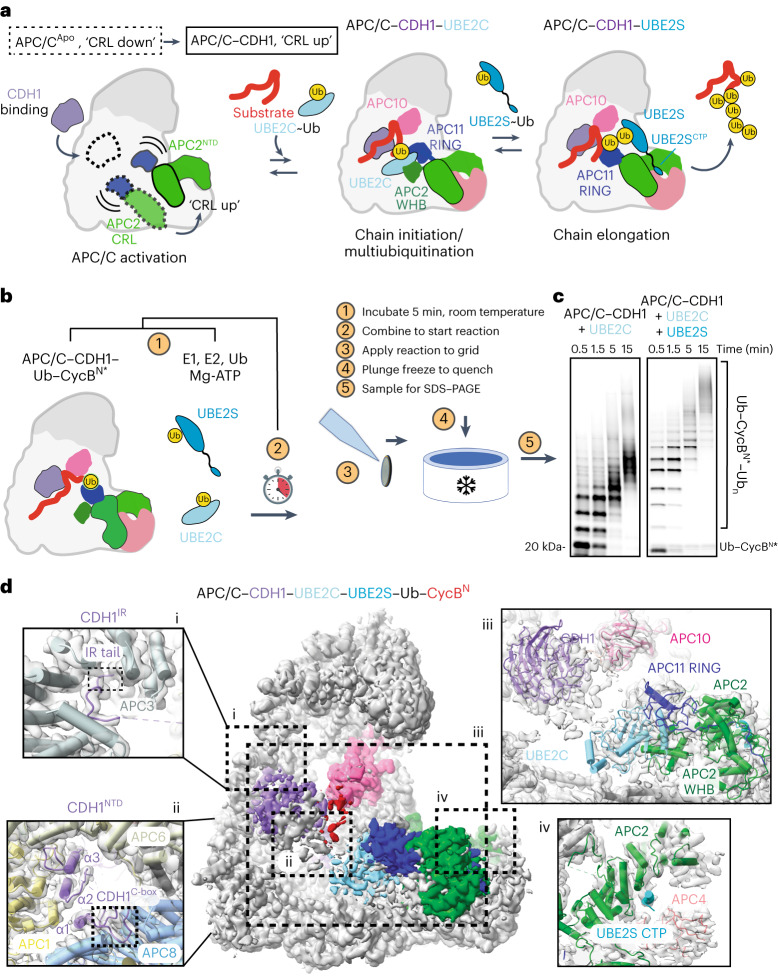
TR-EM reveals structure of APC/C–CDH1–UBE2C–UBE2S^CTP^ during substrate polyubiquitination. **a**, Cartoon representation of reaction cycle carried out by the APC/C as it ubiquitinates substrates. The transition from the ‘CRL (cullin–RING) down’ inactive conformation to the ‘CRL up’ active conformation is facilitated by coactivator binding. Initiation of ubiquitination on target substrates is carried out by the recruitment of UBE2C~Ub by the APC2 WHB (winged-helix B) and APC11 RING domains. UBE2S elongates chains initiated by UBE2C. **b**, Overview of TR-EM approach. Two mixtures containing the reaction components are incubated at room temperature (1). The mixtures are combined to start the reaction (2). Samples are taken from the reaction at timepoints indicated and applied to a grid (3) that is then plunge frozen (4) and imaged using cryo-EM. Representative samples were taken at the time the grids were plunge frozen for SDS–PAGE (5), shown in **c**. **c**, Fluorescent monitoring of an SDS–PAGE gel showing APC/C^CDH1^-E2 (UBE2C or both UBE2C and UBE2S)-dependent substrate modification at each timepoint for which a grid was frozen. At least three experimental replicates were repeated to optimize conditions. Uncropped gel image available in source data. **d**, 3D reconstruction of particles from the filtered APC/C–CDH1–UBE2C–UBE2S dataset at 3.5 Å showing the active catalytic architecture assembled. Atomic models of key subunits and ubiquitination components fitted into the cryo-EM density, including CDH1 bound to the APC/C scaffold (i and ii), UBE2C clamped by the APC2 WHB and APC11 RING domains (iii), and the UBE2S CTP bound to the groove formed by APC2 (green) and APC4 (light pink) (iv). Source data

Substrate ubiquitination requires Ub to be passed along a cascade of E1, E2 and E3 enzymes^14^. E3 enzymes serve as a hub for substrate recognition and directly mediate the transfer of Ub from the E2 to the substrate^15^. A key Ub ligase known as the anaphase-promoting complex/cyclosome (APC/C) drives the cell cycle by recognizing important substrates, for example, cyclin B and securin, and ubiquitinating them to mark them for proteasomal degradation^16,17^. Degradation of such substrates controls cell cycle timing, including mitosis and G1 maintenance^18,19^. A key determining factor in the timing of APC/C substrate degradation is the efficiency of Ub signal formation on the substrate and whether it occurs in a processive or distributive manner^8,9,11,20^. This Ub signal can vary through modification of numerous lysines on the substrate or through different Ub linkages (for example, K11, K48, K63 or K11/K48 branched chains)^21–23^. During a processive ubiquitination cycle, the substrate binds to APC/C coactivators (CDC20 in mitosis and CDH1 in interphase) and APC10 (refs. ^8,24,25^). The binding of the coactivator elicits a conformational change in the cullin–RING (CRL) subunits APC2–APC11, which promotes the binding of the first E2, UBE2C/UBCH10, that adds Ub directly to the substrate (Fig. 1a)^26–28^. UBE2C can either continue to modify substrate lysines or make short chains^21,29–31^. A second E2, UBE2S, extends the K11-linked Ub chains on the substrate^32–34^. These reactions can all occur during a single substrate-binding event while the E2s require multiple rounds of transient binding and catalysis to modify the substrate.

A proteasome degradation signal is typically regarded as a substrate that is modified with four or more Ubs^35^. A structure of an E3 building such signal remains elusive. So far, the structures of APC/C with either UBE2C or UBE2S, and other E3–E2 pairs, have mostly relied on chemical crosslinking approaches to lock the dynamic enzymes in place^23,27,36–40^. These studies allow for high-resolution structural determination of active catalytic architectures but limit our understanding of the conformational landscape and driving forces of E3-mediated polyubiquitination. A single molecule study of APC/C-dependent polyubiquitination revealed that substrate-linked Ub(s) enhance the processivity of the ubiquitination reaction by improving the substrate binding affinity or processive affinity amplification^7^. However, the precise coordination of Ub-binding sites, of E2 binding and of Ub transfer by the APC/C or any E3 to build a proteasome degradation signal remains largely undescribed.

In this Article, we performed time-resolved cryo-EM (TR-EM) experiments to watch the APC/C in action and understand its structural and biophysical properties during substrate polyubiquitination. By combining TR-EM with in vitro ubiquitination assays and total internal reflection fluorescence (TIRF) microscopy, our work provides the framework for a sculpted energy landscape, where particularly low-energy (highly populated) intermediates are not observed along the reaction path of APC/C-dependent polyubiquitination. In general, macromolecular machines such as the APC/C coordinate dynamic equilibrium within flexible domains with stochastic binding of E2s to carry out their activity. These types of molecular systems therefore exhibit highly multimodal heterogeneity. We show that the APC/C couples the thermal noise power of its flexible domains with transient stabilizing interactions and with the emerging Ub-modified substrate to drive processivity. Allosteric changes upon binding of coactivators and the chain elongating E2 UBE2S potentiate this processivity. Together, these interactions allow us to create a model for how APC/C builds a proteasomal degradation signal.

## Results

### Structure of the APC/C actively ubiquitinating a substrate

The APC/C and its E2s can perform a vast number of different ubiquitination reactions^21–23,29–34,41^. Trapping each of these individual structural states has required detailed biochemical knowledge about the system to stabilize the reaction intermediates^23,27,36–38,42^. Given the multiple substrate and Ub lysine target sites, the number of potential ubiquitination states that would require artificial trapping is huge. Furthermore, generating structures of later stages of substrate ubiquitination to help define the determinants of processivity would require complex protein engineering to form different Ub chain linkage types. We therefore sought to solve structures of the APC/C and its E2s performing polyubiquitination over a time course to examine its conformational heterogeneity, identify unknown states of polyubiquitination and uncover interactions between the substrate-linked Ub and the APC/C machinery.

**Extended Data Fig. 1:**
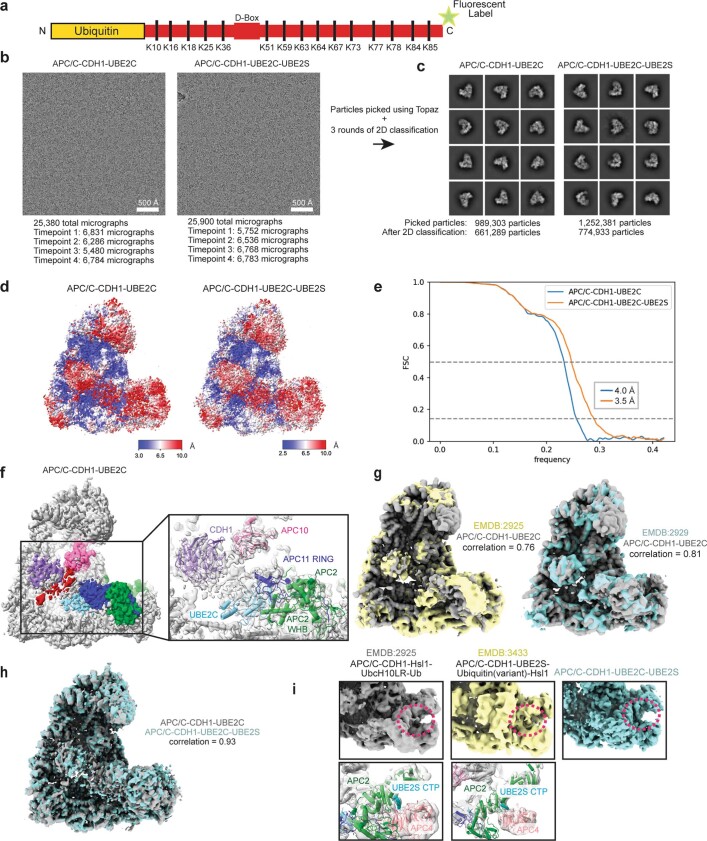
Processing and fitting of the APC/C from the TR-EM datasets. **a** – Diagram depicting domain map of Ub-CycB^N^*. **b, c** – Cryo-EM data processing workflow showing representative micrographs from the APC/C-CDH1-UBE2C and APC/C-CDH1-UBE2C-UBE2S datasets (B). Particle detection was optimized using Topaz^59^ and filtered using 3 rounds of 2D classification. Representative 2D projections from the final 2D classification step show the APC/C in multiple orientations (C). **d** – Local resolution map of the consensus refinements from the datasets. **e** – FSC curves showing resolution of the 3D density maps from the consensus refinement from the APC/C-CDH1-UBE2C and APC/C-CDH1-UBE2C-UBE2S datasets. Lines show the 0.143 gold-standard and 0.5 cut-off with values denoting resolution at 0.143 cut-off. **f** – Similar to Fig. 1d, fitted model of APC/C-CDH1-UBE2C. **g** – Overlay of APC/C-CDH1-UBE2C electron density map from this study with published maps from previous studies. Values shown indicate map-to-map correlation of maps from previous studies (EMD-2925 and EMD-2929) with APC/C-CDH1-UBE2C from this data after gaussian filtering^27,38^. **h** – Overlay of electron density maps from the APC/C-CDH1-UBE2C and APC/C-CDH1-UBE2C-UBE2S consensus refinements, value shown indicates map-to-map correlation. **i** – Models showing UBE2S-CTP bound to the APC2/APC4 groove in previously described datasets (EMD-2925; EMD-3433)^23,27^ and here in the APC/C-CDH1-UBE2C-UBE2S dataset.

To generate TR-EM samples of the APC/C during substrate ubiquitination, we preincubated two mixtures, one containing the APC/C, CDH1 and substrate, and a second containing the E1, UBE2C, Mg-ATP and Ub (Fig. 1b). For a substrate, the fluorescently labeled CycB N-terminus (Ub-CycB^N^*) containing a fused Ub was used to ensure that additional observed interactions resulted from the formation of polyubiquitination rather than the initial substrate priming step (Extended Data Fig. 1a). To examine the impact of UBE2S on the structural landscape of APC/C^CDH1^, a second reaction was performed that included UBE2C and UBE2S. The two mixtures were combined to start the reaction, and samples were taken from the reaction at specific timepoints (0.5, 1.5, 5 and 15 min), applied to a grid and plunge frozen. Visualization of the reaction on sodium dodecyl sulfate–polyacrylamide gel electrophoresis (SDS–PAGE) showed an expected difference between the two time courses, with the presence of both E2s resulting in longer Ub chains than UBE2C alone (Fig. 1c). Both reactions showed differences in levels of Ub modification between the initial and later stages of the reaction. Single-particle electron microscopy (EM) data were then collected from each grid at each timepoint (Extended Data Fig. 1b).

The data collected on a Titan Krios resulted in 25,380 and 25,900 movies for the reaction mixtures containing APC/C^CDH1^–Ub–CycB^N^–UBE2C and APC/C^CDH1^–Ub–CycB^N^–UBE2C–UBE2S, respectively (Extended Data Fig. 1b,c). After multiple rounds of classification to filter each dataset down to ~600,000–700,000 particles, we were able to solve high-resolution consensus structures (3–4 Å) for each dataset (Extended Data Fig. 1d–f and Table 1). Because of prior work, we were able to build the structure of APC/C interacting with its coactivator CDH1 and UBE2C for both datasets (Fig. 1d and Extended Data Fig. 1f). The coactivator N- and C-terminal tails are also resolved in their respective binding sites, with the CDH1 IR tail localized on APC3 and the CDH1 C-box bound to APC8 (Fig. 1d, i and ii) (ref. ^27^). The D-box of CycB is seen bound to the CDH1–APC10 groove and UBE2C is clasped by the APC11 RING and APC2 WHB (Fig. 1d, iii) (refs. ^23,27,38^). However, a key difference between these two structures (Fig. 1d and Extended Data Fig. 1f) and previous work is that UBE2C is not artificially trapped through chemical crosslinking or through genetic fusion to either APC11 or the UBE2S C-terminus^23,27,38^. Density for the UBE2S C-terminal peptide (CTP) is visible in the groove formed by APC2–APC4 in the dataset containing both E2s (Fig. 1d, iv) (refs. ^23,27^). Therefore, our structures validate prior work that relied on chemical crosslinking with UBE2C and UBE2S and reveal structures of an E3–E2–E2 complex actively ubiquitinating a substrate, indicating that both E2s can bind the APC/C simultaneously^23,27,38^.

When comparing the structure of APC/C–CDH1–UBE2C from this study to those of the same complex from two previous studies (EMD-2925 and EMD-2929), the structures were similar, with map-to-map correlations of either 0.76 or 0.81 (Extended Data Fig. 1g)^23,27,38^. When we compared the structures of APC/C–CDH1–UBE2C and APC/C–CDH1–UBE2C–UBE2S generated from the two datasets in this study, we observed a map-to-map correlation of 0.93 (Extended Data Fig. 1h). In other words, the reaction that contained UBE2S did not reveal extra density for the UBE2S catalytic core. The observed density for the UBE2S^CTP^ in our APC/C–CDH1–UBE2C–UBE2S structure did agree with structures from previous studies where it was resolved (Extended Data Fig. 1i). Ultimately, this is consistent with the previous kinetic work suggesting that the UBE2S^CTP^ provides the affinity for UBE2S to bind to the APC/C, suggesting that the intrinsic kinetic differences (on and off rates) between E2 and E3 interactions are a potential limiting factor to solving structures of E3–E2 pairs actively ubiquitinating a substrate^23,27,42,43^. These differences could be due to either binding interactions or the type of ubiquitination occurring, that is, ubiquitinating the target directly versus building a polyubiquitin chain.

### Total heterogeneity reveals a sculpted energy landscape

**Extended Data Fig. 2:**
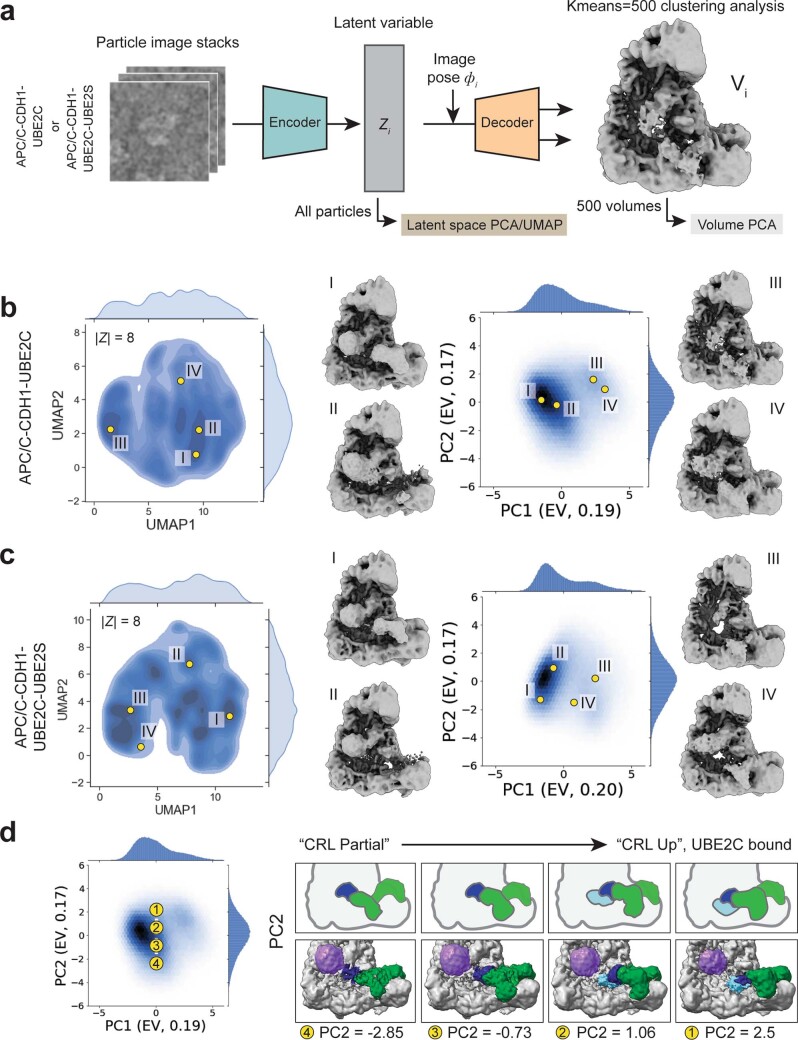
Heterogeneity analysis and classification of particles from the TR-EM datasets using cryoDRGN and PCA. **a** – Diagram depicting the cryoDRGN data processing workflow. Particle images are mapped into a continuous low-dimensional vector space, producing a latent embedding, z. A volume decoder can then reconstruct a unique 3D volume given any value of z, for example a particle’s mapped location in latent space. Principle Component Analysis (PCA) can be applied to either the set of particle latent embeddings for a dataset or a set of reconstructed 3D volumes to map the heterogeneity of the particles or volumes in a continuous manner. **b**, **c** – UMAP (left) and PCA (right) depiction of the latent space with representative volumes shown at indicated locations, for APC/C-CDH1-UBE2C (B) and APC/C-CDH1-UBE2C-UBE2S (C), explained variance (EV) noted in parentheses. **d** – PC trajectory along the second component showing the transition between a ‘CRL Partial’ to ‘CRL Up, E2 Bound’ state. Values in parentheses denote explained variance (EV). Densities CDH1, APC2, APC11, and UBE2C are colored as purple, green, blue, and cyan, respectively.

To provide further insight into the conformational landscape of the APC/C and its E2s and understand processive polyubiquitination, we examined the structural heterogeneity of the APC/C during catalysis in the two datasets using cryoDRGN (Deep Reconstructing Generative Networks)^3^. The cryoDRGN method learns a mapping of particle images into a low-dimensional continuous vector space, termed the latent space, and an associated neural network volume representation that can reconstruct a 3D density volume from any point in this latent space, allowing for the visualization of a unique 3D map for each particle (Extended Data Fig. 2a). The full dataset’s latent space representation can be visualized in 2D using dimensionality reduction techniques such as principal component analysis (PCA) and uniform manifold approximation and projection (UMAP)^44^. The distribution of structures can be further analyzed by *k*-means clustering on the latent space representation, with particles classified into various states based on their cluster assignments. For each of the two time courses, particles across all timepoints were combined and used to train the neural network. UMAP visualization of the latent space for both time courses revealed a continuous, featureless surface, suggesting a convoluted landscape with high degrees of overlap between the distribution of structures across time points (Extended Data Fig. 2b,c). A comprehensive sampling of 500 structures from the latent space at the *k*-means cluster centers (*k* = 500) showed largely similar compositional and conformational variability between the datasets and recapitulated several known APC/C conformations (Extended Data Fig. 2b,c).

**Fig. 2:**
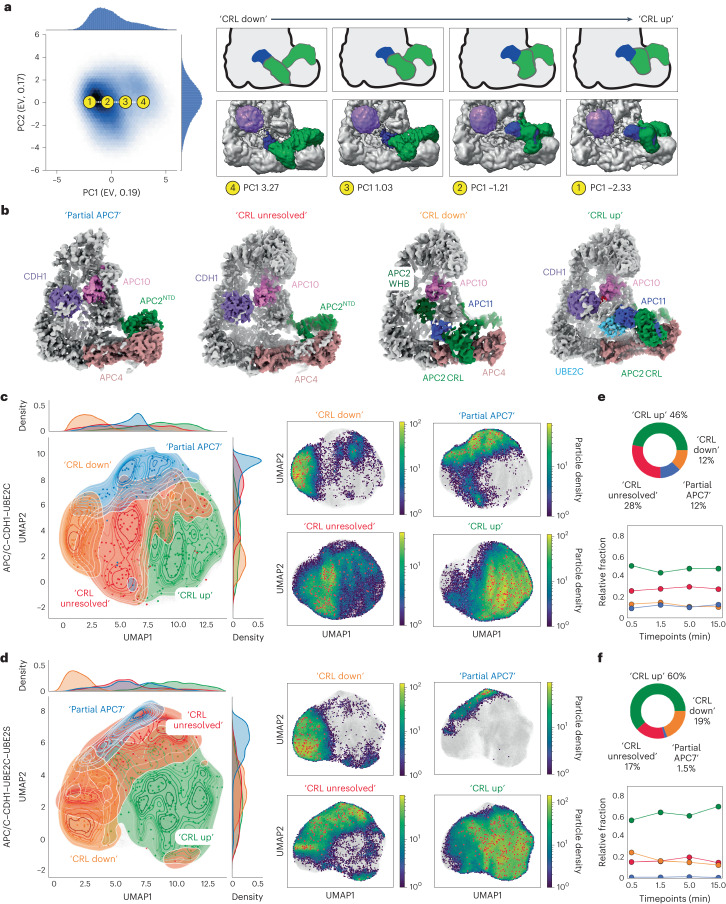
Reconstruction of a functional conformational landscape of APC/C–CDH1-mediated polyubiquitination using cryoDRGN. **a**, PCA representation of the particle distribution in latent space with explained variance (EV) noted in parentheses. The first component captures the continuous conformational changes the APC/C undergoes in the ‘CRL down’ to ‘CRL up’ transition. Density for APC2, APC11 and CDH1 are shown at indicated points as green, blue and purple, respectively. **b**, 3D models generated using homogeneous 3D refinement of each set of major state particles. Density maps are colored by proximity to the subunit indicated as modeled using rigid body fitting. **c**,**d**, Left: UMAP representation of the particle distribution in latent space clustered using *k*-means (*k* = 500) clustering and then classified into the four major APC/C states present within the APC/C^CDH1^–UBE2C (**c**) and APC/C^CDH1^–UBE2C–UBE2S (**d**) datasets. Points denote cluster centers where volumes were generated. Right: subplots of particle distributions separated by major states. Particle distributions for each class are colored by their local density overlayed onto the particle distribution for the entire dataset (gray). **e**,**f**, Top: charts show distribution of particles across major states for the APC/C^CDH1^–UBE2C (**e**) and APC/C^CDH1^–UBE2C–UBE2S (**f**) datasets. Bottom: line graphs show change in relative fraction of each major state over time in the two datasets.

Trajectories generated along the principal components of the latent space captured the broad conformational changes of the APC/C. For example, the trajectory along the first component (PC1) shows the continuous motion through which the APC2 CRL domain (the C-terminus of APC2) transitions from the ‘CRL down’ to the ‘CRL up’ conformation (Fig. 2a). Along the second component (PC2), the APC2 CRL can be visualized transitioning from an intermediate, partially raised ‘CRL up’ conformation to the fully raised ‘CRL up’ conformation with clear density for UBE2C present (Extended Data Fig. 2d and Supplementary Video 1).

**Extended Data Fig. 3:**
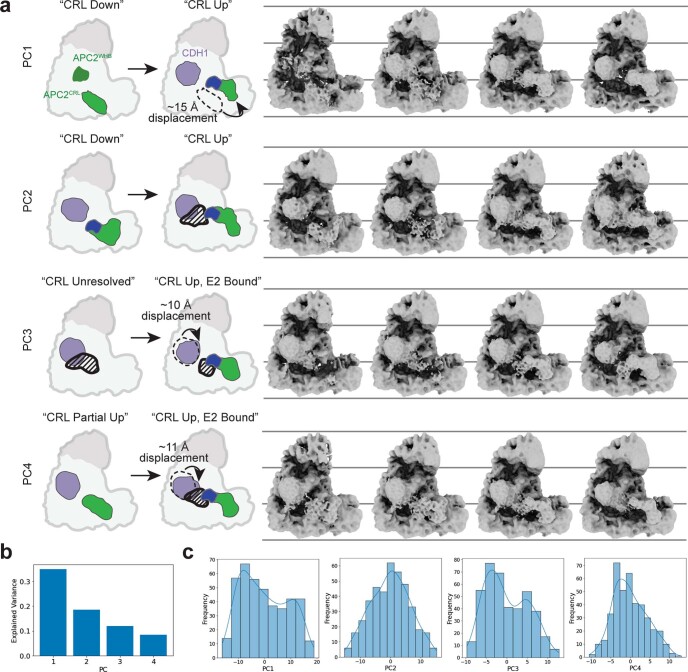
Movement of the APC/C during catalysis, shown in cryoDRGN volume PCA trajectories. **a** – Volume PCA trajectories showing conformational changes along first four components to visualize displacement of CDH1 and the APC2 CRL along continuous conformational trajectories. Density changes localized near CDH1 and at the APC/C actives site shown in black stripes. **b** – Expected variance plotted for the first four components shown in A. **c** – Histograms showing distributions of volumes along the PCs shown in A.

By applying PCA on the 500 volumes generated at the *k*-means cluster centers, we were able to uncover interesting aspects of conformational variability of the APC/C (Extended Data Fig. 3). Along the first volume PC trajectory, we can trace the binding of the coactivator together with the simultaneous displacement of the APC2 CRL domain from the ‘down’ to the ‘up’ conformation, resulting in a ~15 Å displacement. The second volume PC trajectory follows a CDH1-bound state where the CRL is partially in the ‘up’ conformation and undergoes an ~11 Å displacement to the ‘CRL up’ conformation. The third and fourth PCs map heterogeneity at the coactivator and the catalytic core, with CDH1 displacements of ~10 and ~11 Å visualized, respectively. Changes in density present near the coactivator and contacts made and broken between the components involved in the catalytic core (CDH1, UBE2C, APC11, APC2^CRL^ and APC10) are also visualized, demonstrating the power of cryoDRGN for visualizing complex heterogeneity. Extra density, particularly near the coactivator and UBE2C visualized in PC3 and PC4, suggests heterogeneity at the catalytic core due to the presence of substrate and Ub.

**Extended Data Fig. 4:**
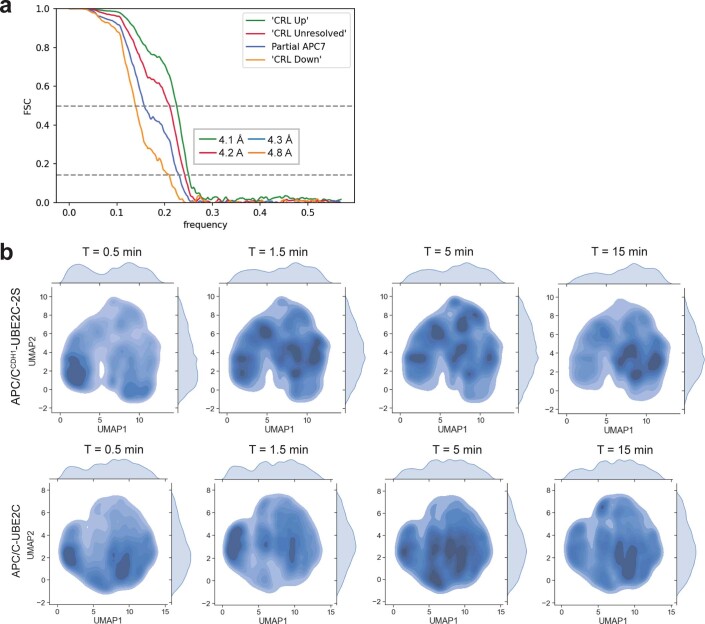
FSC curves and UMAP particle distributions over TR-EM datasets. **a** – FSC curves depicting the resolution of the consensus 3D refinements shown in Fig. 2b. Lines show the 0.143 gold-standard and 0.5 cut-off with values denoting resolution at 0.143 cut-off. **b** – UMAP visualization of the particle distributions in the latent space plotted separately by timepoint.

A systematic analysis of the 500 cryoDRGN volumes revealed four major conformational states: APC/C with the APC2^CRL^ domain in a ‘CRL down’ conformation, an APC/C^CDH1^-‘CRL up’ conformation, and two categories of states where either APC7, located at the top of the APC, or the APC2 CRL–APC11 RING domains showed high degrees of variability (labeled ‘Partial APC7 and ‘CRL unresolved’, respectively) (Fig. 2b and Extended Data Fig. 4). While the biological relevance of the ‘Partial APC7’ and ‘CRL unresolved’ states is currently unclear, some differences within these states can be noted. For example, the classes where APC2–APC11 are unresolved all contained CDH1. Additionally, extra density was observed around the coactivator and the APC2 N-terminal domain remained clearly resolved, suggesting the potential for dynamics within the APC2–APC11 domains that are not captured by our methods.

The mapping of the four major states onto the UMAP visualization of the particle distribution in the latent space revealed a convoluted landscape with some degree of overlap between the states (Fig. 2c,d). Interestingly, the mapping of the distribution of the states relative to one another was preserved in the landscapes of both datasets. Between the two datasets, the one containing only UBE2C showed higher fractions of particles in the ‘Partial APC7’ and ‘CRL unresolved’ states, suggesting a potential role for UBE2S in stabilizing these domains or the APC/C overall (Fig. 2e,f). Both datasets showed similar levels of ‘CRL down’ particles. Overall, the distribution of particles within the states changed relatively little across the timepoints, suggesting an energy landscape void of new highly populated APC/C intermediates (∆∆*G* < 2 k_B_T) in the mixture in any of the possible conformational states at a given time and regardless of the extent of overall substrate ubiquitination. This observation suggests that polyubiquitination is not a process with large energy barriers, and instead the reactions are stochastic, with thermal noise and allostery due to the simultaneous binding of multiple components driving the conformational states. Thus, directionality in the processivity of the reaction needs to be introduced by different means than energy barriers.

### UBE2C association with APC/C is increased by CDH1 and UBE2S

**Fig. 3:**
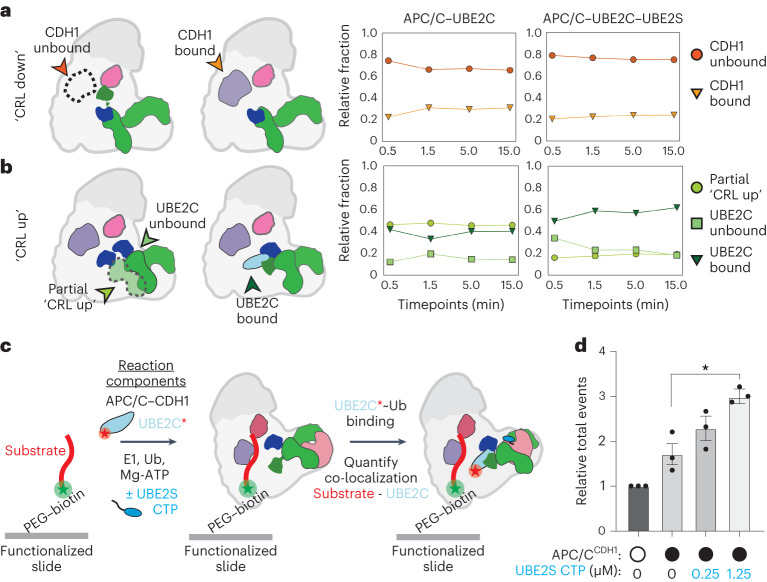
UBE2S CTP enhances the APC/C ‘CRL up’ state, increasing the association of UBE2C~Ub with APC/C–CDH1. **a**,**b**, Quantification of substates within the APC/C ‘CRL up’ and ‘CRL down’ major states assigned from 500 sampled cryoDRGN volumes. ‘CRL down’ clusters partition into a majority of particles where CDH1 is absent (~80%). The ‘CRL up’ particles contain a minor subset of particles where the CRL is only partially in the ‘up’ position (**a**). Addition of UBE2S increases the relative number of particles with density attributed to UBE2C present in the clusters compared to the absence of UBE2S (**b**). **c**, Schematic of TIRF microscopy substrate immobilization and the localization of ubiquitination reaction components. Alexa488-labeled CycB^N^ is immobilized to a neutravidin-functionalized slide through an N-terminal, biotinylated Avitag (captured using a 488 nm laser; substrate channel). APC/C–CDH1 will localize to the substrate and subsequently recruit fluorescent UBE2C–JF549 (captured using a 561 nm laser; UBE2C channel) and a UBE2S^CTP^ peptide that binds the APC2/APC4 groove. Thus, the number of binding events in the UBE2C channel correlates to UBE2C localizing to APC/C–CDH1–substrate. **d**, Titrating the UBE2S^CTP^ increases the recruitment of UBE2C to APC/C–CDH1, localized to substrate at the slide surface. Quantification of the total number of UBE2C binding events (Extended Data Fig. 5f) from three independent experiments is shown (see Supplementary Video 2 for representative movies and Extended Data Fig. 5g,h for single molecule binding event montage and representative traces). Error bars: standard error of the mean. Data are normalized to the control where APC/C-CDH1 is absent. Experiments were compared using one-way analysis of variance (*P = 0.0176). Source data

**Extended Data Fig. 5:**
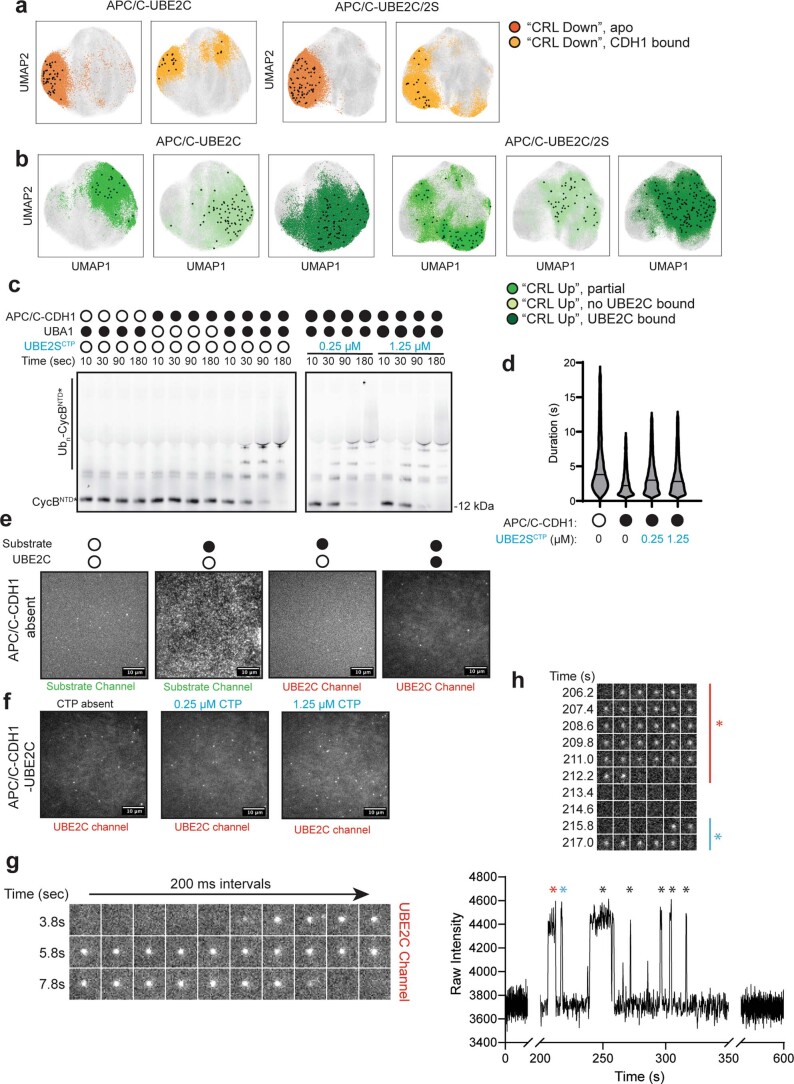
Development of TIRF-based APC/C-dependent substrate ubiquitination assay following fluorescently-labeled UBE2C. **a, b** – UMAP representation showing the particle distribution of the sub-states in the ‘CRL Down’ (A) and ‘CRL Up’ (B) major states. **c** – TIRF microscopy movies capture substrate-E2 interactions prior to complete substrate depletion. Experimental mixtures for the given conditions were split evenly between microscopy flow cells for data capture (Fig. 3d) and a ubiquitination reaction with 50 nM Biotin-CycB^N^*-A488 spiked in, shown above, that was ran on an SDS-PAGE gel and fluorescently scanned. The banding of the ubiquitinated substrate is distorted due to the high BSA concentration needed for microscopy. The experiments were performed independently three times. **d** – Duration of detected binding events in seconds across multiple experiments and with increasing concentration of UBE2S CTP. **e** – There is minimal background signal from slide functionalization (left) and robust signal from immobilized substrate (center left) in the Substrate channel, as well as minimal background fluorescence from the substrate in the UBE2C channel (center right). The addition of UBE2C to immobilized substrate without APC/C-CDH1 shows minimal non-specific UBE2C binding (right). **f** – Example images showing UBE2C binding events upon addition of APC/C-CDH1 and increasing levels of UBE2S CTP. **g** – Montage showing representative time series of a UBE2C binding event in the UBE2C channel at 200 ms intervals. **h** – Montage (top) depicting duration of individual binding events in experiments described in (C) indicated by red or blue asterisk. Trace of raw intensity values (bottom) showing individual UBE2C binding events; red or blue asterisks denote corresponding binding events shown in montage (top). Source data

Further analysis of the volumes from *k*-means clustering major states revealed additional characteristics about APC/C conformational dynamics. The ‘CRL down’ major state clusters could be subdivided into two substates: an apo state that showed no density near APC10 that could be attributed to CDH1 and a small minority of clusters that did show density consistent with CDH1 bound (Fig. 3a and Extended Data Fig. 5a). The much higher level of ‘CRL down’ in the apo ‘CDH1 unbound’ state is consistent with previous data showing that coactivator binding mediates the transition from the ‘CRL down’ to the ‘CRL up’ states^26,28^. For the ‘CRL up’ state, where we expect that allostery through binding is a directing force, we examined if the composition of these states is altered by the presence of UBE2S, as suggested previously^45^. When UBE2S is present, the UBE2C-bound fraction is slightly increased at each timepoint (Fig. 3b and Extended Data Fig. 5b). In the reaction without UBE2S, UBE2C is present in ~40% of the particles. When UBE2S is added, UBE2C is present in ~50–60% of the particles over the time course. This finding suggests that UBE2S improves the association of UBE2C with the APC/C and potentiates multiubiquitination.

To investigate cooperative interactions between the APC/C^CDH1^-associated E2s on a single-molecule level, we developed a TIRF microscopy-based system. Briefly, a fluorescently labeled, biotinylated substrate (biotin–CycB^N^*) was immobilized within flow chambers functionalized with neutravidin (Fig. 3c). UBE2C labeled with JaneliaFluor549 (UBE2C-JF549) was mixed with ubiquitination reaction components, including unlabeled APC/C, then added to the flow cells immediately before data acquisition. These tagged and labeled reagents retained their functionality in substrate ubiquitination reactions (Extended Data Fig. 5c). Within the flow chamber, APC/C^CDH1^ will bind the immobilized substrate and recruit UBE2C–JF549 from solution to the slide surface for ubiquitination. Therefore, fluorescence signal events at the slide surface provide a readout of UBE2C–JF549 recruitment by APC/C^CDH1^. We observed minimal background fluorescence from the functionalization process, robust signal from immobilized biotin–CycB^N^*, and minimal signal from the fluorescent substrate in the UBE2C channel, indicating that our TIRF system allows for a clear delineation between substrate and E2 signals at the slide surface (Extended Data Fig. 5d–h).

Next, we sought to assess how the UBE2S^CTP^ impacts UBE2C recruitment by titrating a UBE2S^CTP^ 18mer peptide into the reaction. Addition of the UBE2S^CTP^ substantially increased the total events of UBE2C localizing to the slide surface, indicating that the UBE2S^CTP^ enhances UBE2C recruitment to APC/C^CDH1^-bound substrate (Fig. 3d, Extended Data Fig. 5f and Supplementary Video 2). This result supports prior work that UBE2S is capable of stimulating UBE2C activity through its unique binding arrangement on the APC/C^45^. Altogether, we demonstrate coordination between UBE2C and the UBE2S^CTP^ at APC/C^CDH1^ and present a flexible system to study substrate-dependent interactions with single-molecule resolution. Furthermore, this system corroborates our structural data that UBE2S assists in stabilizing the ‘CRL up’ conformation, permitting UBE2C to bind the APC/C.

### APC/C^CDH1^–UBE2C structures mediating polyubiquitination

**Extended Data Fig. 6:**
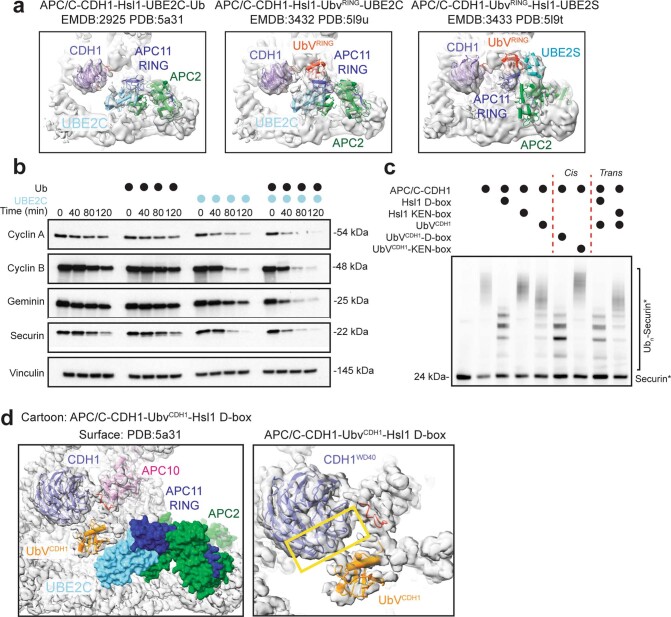
Previous artificially-trapped APC/C structures mimicking substrate ubiquitination. **a** – Fitted cryoEM models from previous studies of the APC/C showing monoubiquitination (left), multi-ubiquitination (center), and polyubiquitination (right)^23,38^. An Hsl1 truncation harboring its KEN- and D-box was used as a substrate where a lysine was replaced with cysteine and crosslinked to the active site of UBE2C to mimic substrate priming (left). A similar Hsl1 truncation was genetically fused to the UbV^RING^, purified, and crosslinked to UBE2C and Ub to mimic multi-monoubiquitination (center). For chain elongation, a UbV^RING^-Hsl1 D-box chimera harboring a K11C substitution was crosslinked to the active site of UBE2S (right). **b** – Similar to Fig. 4e, immunoblotting of APC/C-dependent substrate degradation in mitotic HeLa cell extracts reveals that this process is not inhibited by the addition of excess Ub, in contrast to the UbV^CDH1^. The experiments were performed independently three times. **c** – Similar to Fig. 4f, Securin ubiquitination is inhibited more strongly by the UbVCDH1-Hsl1 D-box fusion, as monitored by fluorescent scanning of APC/C-CDH1-UBE2C-dependent ubiquitination reactions. The experiments were performed independently three times. **d** –Cryo-EM map of APC/C-CDH1-UbV^CDH1^-Hsl1 D-box rigid body fitted with a surface representation model of UBE2C bound to APC2 (green)/APC11(blue). Left, UbV^CDH1^ (orange) is in position to receive Ub from UBE2C (cyan). Right, cryo-EM map fitted with atomic models of CDH1 (purple) and UbV^CDH1^ (orange)-Hsl1 D-box (red). Yellow rectangle indicates the KEN-box binding site of CDH1, similar to where UbV^CDH1^ binds. Uncropped gels representative of n = 3 independent experiments for C available in source data. Source data

So far, most structures of RING E3s bound to an E2 involve an unmodified substrate mimicking substrate priming or a monoubiquitinated substrate to mimic monoubiquitination, multiubiquitination (targeting multiple lysines on a substrate), or specific chain elongation (Extended Data Fig. 6a)^23,27,36–40^. Since our datasets involve the entire polyubiquitination process, we rationalized that localized classification approaches would help us identify APC/C structures further along polyubiquitination than ever before. This processing resulted in unexpected insights into RING E3–E2-dependent polyubiquitination.

**Fig. 4:**
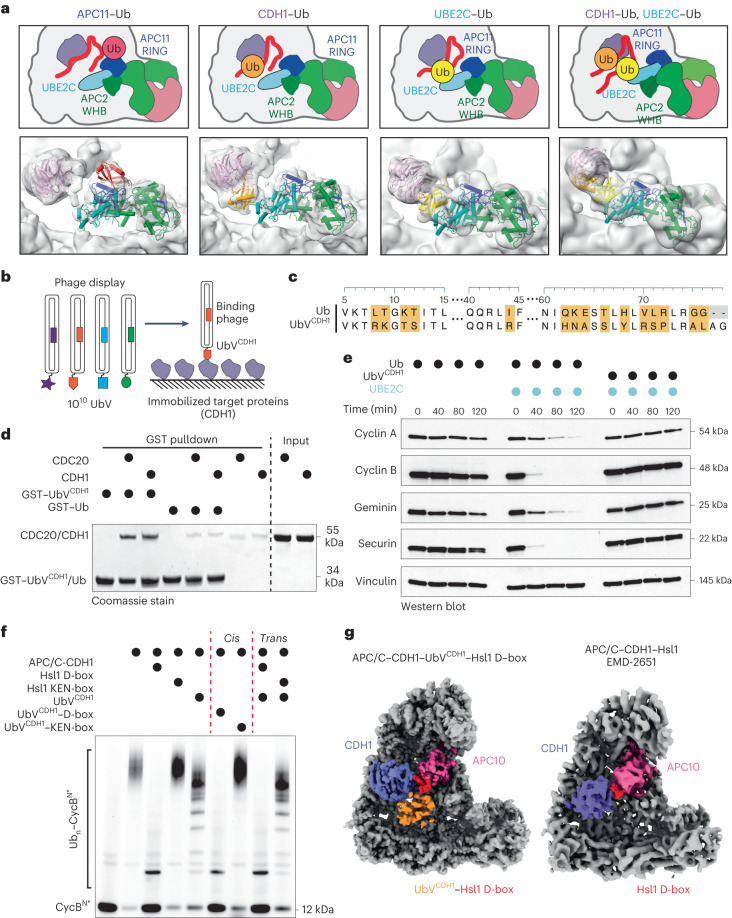
Analysis of active APC/C-dependent ubiquitination architecture reveals unexpected Ub binding modes. **a**, Focused 3D classification on CDH1, UBE2C and APC2/APC11 using a mask to generate structures of the active APC/C. Discrete states were found showing a Ub interacting with the known APC11 RING exosite (left) and forming contacts with CDH1 (center left) and UBE2C (center right). A class of particles also contained a structure with two Ubs making simultaneous contact with the coactivator and UBE2C simultaneously (right). **b**, Phage display selected a UbV (UbV^CDH1^) that binds to APC/C coactivators. **c**, Sequence alignment of Ub and UbV^CDH1^ with differences highlighted. **d**, Coomassie-stained SDS–PAGE gel showing binding of GST–UbV^CDH1^ towards APC/C coactivators (CDH1 and CDC20), but not GST–Ub, after GST pulldown. *n* = 3 independent experiments. **e**, Degradation of APC/C substrates Cyclin A, Cyclin B, Geminin and Securin in mitotic HeLa cell extracts was inhibited by the addition of UbV^CDH1^, but not Ub, as monitored by immunoblotting. *n* = 3 independent experiments. **f**, Attachment of the Hsl1 D-box, but not the Hsl1 KEN-box, to UbV^CDH1^ potentiates its inhibition of APC/C–CDH1–UBE2C-mediated substrate ubiquitination, monitored in three independent experiments by fluorescent scanning of an SDS–PAGE gel. **g**, Left: the cryo-EM structure of APC/C-CDH1 bound to the UbV^CDH1^(orange)–Hsl1 D-box (red) shows the localization of UbV^CDH1^ near the KEN-box-binding site on the CDH1 β-propeller (purple). Right: previously published cryo-EM map of APC/C bound to CDH1 and Hsl1 (EMD-2651) for comparison^28^. Uncropped gels representative of *n* = 3 independent experiments for **d**–**f** available in source data. Source data

Local classification using a mask around the active site resulted in a set of unprecedented structures for APC/C–UBE2C-mediated polyubiquitination. In addition to known interactions, multiple additional Ub interactions were identified. First, a Ub is bound to the RING domain on the opposite side of the UBE2C-binding site (Fig. 4a, left). This finding is particularly interesting as prior work relied on a tight-binding ubiquitin variant (UbV) to observe this interaction^23^. Next, we observed unexpected density consistent with the size and shape of Ub at the coactivator, suggesting a potential Ub-binding site (Fig. 4a, center left). Additional density is observed between the coactivator and the C-terminal region of UBE2C known as the Helix-Turn-Helix (HTH) (Fig. 4a, center right). Intriguingly, a subset of particles contained density where seemingly a di-Ub contacts both the coactivator and the E2 simultaneously (Fig. 4a, right).

As Ub binding can modulate the activity of E2s and E3s, these potential Ub interactions with either APC11 RING, UBE2C or CDH1 could have unappreciated effects on APC/C-dependent ubiquitination. Since we previously identified that Ub can interact with the APC11 RING domain and the HTH from other E2s (refs. ^23,46^), we sought to examine the function of the unexpected Ub-binding site on CDH1. As tight-binding UbVs have been tremendously helpful in disentangling the regulation of the several enzymes of the Ub system^23,47–50^, including the APC11 RING domain, we selected a library of UbVs against both APC/C coactivators, CDH1 and CDC20 (Fig. 4b). This process revealed a UbV (henceforth known as UbV^CDH1^) that was substantially enriched during the selection (Fig. 4c). To validate that UbV^CDH1^ interacts with the coactivators in a manner similar to wild-type Ub, we performed a series of tests. First, a co-pulldown assay found that a GST–UbV^CDH1^ fusion bound both CDC20 and CDH1 better than the GST–Ub fusion (Fig. 4d). Because the binding site of UbV^CDH1^ is potentially in proximity to the KEN-box degron binding site, we expected UbV^CDH1^ to inhibit APC/C-mediated ubiquitination. Consistent with this hypothesis, UbV^CDH1^ inhibited APC/C substrate degradation in mitotic extracts of HeLaS3 cells (Fig. 4e). Conversely, substrate degradation rates were not influenced by the presence or absence of excess Ub upon the addition of UBE2C (Extended Data Fig. 6b).

UbV^CDH1^ was then used as surrogate to determine the structure of APC/C^CDH1^ bound to Ub. To optimize UbV^CDH1^ for structural studies, we tested if UbV^CDH1^ could be fused to either the KEN-box or D-box of Hsl1, an APC/C substrate, to further strengthen its interaction with the APC/C. As expected, the UbV^CDH1^–Hsl1 D-box fusion markedly inhibited APC/C-mediated substrate ubiquitination compared to either the UbV^CDH1^ alone or the UbV^CDH1^-Hsl1 KEN-box fusion (Fig. 4f and Extended Data Fig. 6c). Therefore, APC/C^CDH1^ bound to UbV^CDH1^–Hsl1 D-box was subjected to cryo-EM. As expected, the Hsl1 D-box was bound between APC10 and CDH1 and the UbV^CDH1^ was bound to CDH1 near the KEN-box binding site (Fig. 4g and Extended Data Fig. 6d). These data are consistent with the ubiquitination assays showing that the UbV^CDH1^–Hsl1 KEN-box fusion weakly inhibited the APC/C-mediated substrate ubiquitination compared to UbV^CDH1^ alone (Fig. 4f and Extended Data Fig. 6c).

### Multiple Ub-binding sites promote processive ubiquitination

**Fig. 5:**
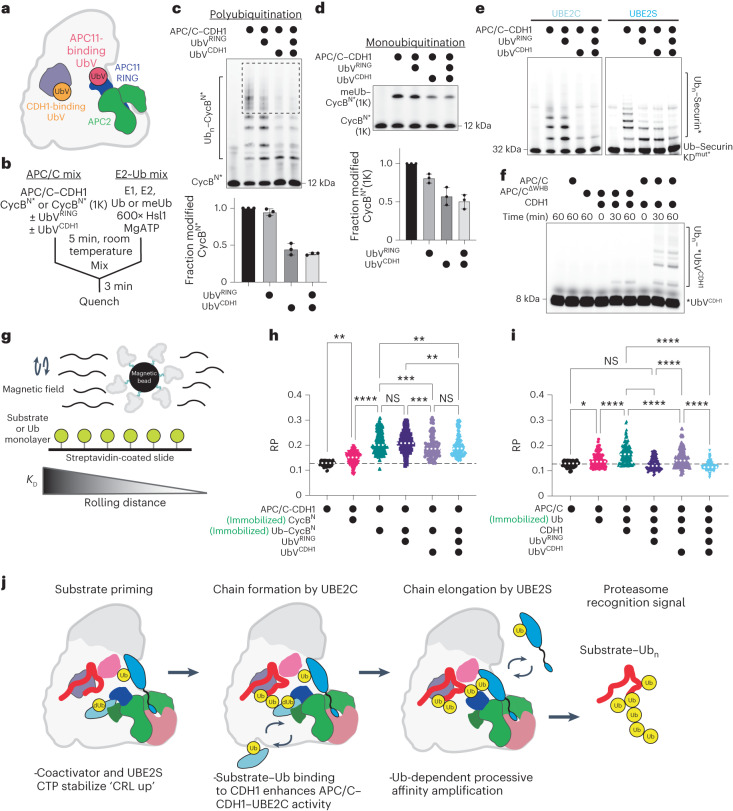
Multiple Ub-binding sites promote processive ubiquitination by APC/C^CDH1^. **a**, Cartoon model showing binding sites of CDH1-binding (UbV^CDH1^) and APC11-binding (UbV^RING^) UbVs. **b**, Workflow for single-encounter experiments. APC/C^CDH1^ and *CycB^N^ containing multiple lysines or a single lysine (1K) are preincubated. The UbVs were added to this mixture. A second mix is prepared containing E1, UBE2C, MgATP, an excess of unlabeled Hsl1, and Ub or methylated Ub (meUb). The mixtures are combined, resulting in fluorescent substrate ubiquitination during a single binding event. **c**, Fluorescent scanning of an SDS–PAGE gel (top) and quantification (bottom) from single-encounter APC/C–CDH1–UBE2C-dependent polyubiquitination reactions with CycB^N^* and Ub. Dashed box indicates the region of CycB^N^* with >4 Ubs quantified in Extended Data Fig. 7a. *n* = 3 independent experiments. Error bars: standard error of the mean. **d**, Fluorescent scanning of an SDS–PAGE gel (top) and quantification (bottom) from single encounter of APC/C–CDH1–UBE2C-dependent substrate priming reactions with CycB^N^*(1K) and meUb. *n* = 3 independent experiments. Error bars: standard error of the mean. **e**, APC/C–CDH1-dependent ubiquitination reactions of Ub–Securin with its KEN- and D-box degrons mutated (Ub–Securin KD^mut^*) using either UBE2C (left) or UBE2S (right), monitored by fluorescent scanning. *n* = 3 independent experiments. **f**, Ubiquitination of fluorescently labeled *UbV^CDH1^ by APC/C–UBE2C is dependent on CDH1 and the APC2 WHB, monitored by fluorescent scanning. *n* = 3 independent experiments. **g**, METRIS setup to monitor binding of APC/C to a biotinylated substrate, Ub–substrate or Ub on a streptavidin-coated surface. Magnetic beads with biotinylated APC/C are subjected to a magnetic field. The movement of individual beads is quantitated to determine a rolling parameter (RP) that correlates with the strength of the interaction. **h**,**i**, Scatter plots quantitating the RP of the APC/C on surfaces containing CycB^N^ or Ub-CycB^N^ (**h**) or Ub (**i**) in the presence of UbV^RING^, UbV^CDH1^ or both. Experiments were compared using one-way analysis of variance (****P ≤ 0.0001, ***P ≤ 0.001, **P ≤ 0.005, *P ≤ 0.05). NS, not significant. **j**, Schematic of APC/C–CDH1–UBE2C–UBE2S-dependent substrate polyubiquitination. The APC/C catalytic architecture and recruitment of UBE2C~Ub (dUb) is influenced by CDH1 and UBE2S CTP binding to drive ubiquitination efficiency. Allosterically driven processivity of ubiquitination is further mediated by stabilizing contacts between multiple substrate-linked Ubs and CDH1 and APC11. Uncropped gels representative of *n* > 3 independent experiments for **c**–**f** available in source data. Source data

**Extended Data Fig. 7:**
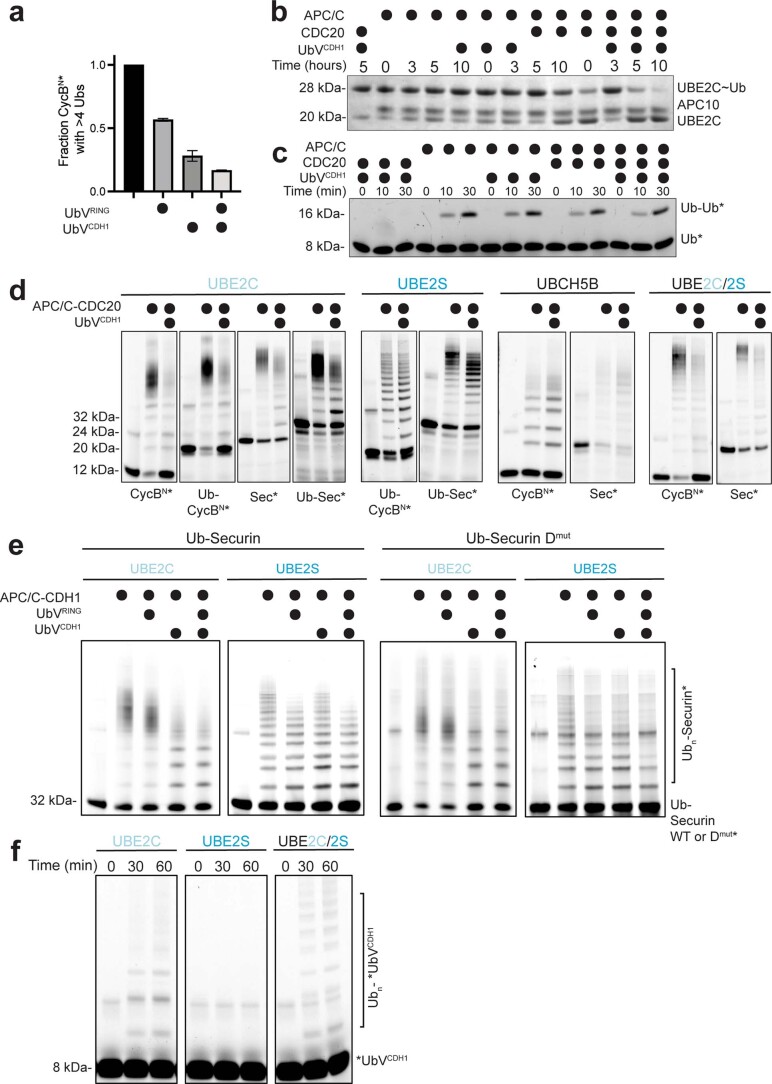
Ub binding to coactivator serves multiple purposes during APC/C-dependent polyubiquitination. **a** – Quantitation of CycB^N^* modified with >4 Ubs from single encounter assays in Fig. 5c. Error bars: SEM. The experiments were performed independently three times. **b** – Substrate-independent, APC/C-dependent hydrolysis of an oxyester-linked Ub from UBE2C C114S is not impeded by the addition of UbV^CDH1^, monitored by Coomassie-stained SDS-PAGE gel. The experiments were performed independently three times. **c** – Synthesis of di-Ub by APC/C-UBE2S is not inhibited by UbV^CDH1^ as revealed by scanning of an SDS-PAGE gel where the acceptor Ub* is fluorescently labeled. The experiments were performed independently three times. **d** – UbV^CDH1^ inhibits APC/C-CDH1-dependent ubiquitination activity of all substrate and E2 combinations tested, monitored by fluorescent scanning. The experiments were performed independently three times. **e** – Ubiquitination of Ub-Securin and Ub-Securin D^Mut^ as shown in Fig. 5c. The experiments were performed independently three times. **f** – Similar to Fig. 5f, ubiquitination of *UbV^CDH1^ requires UBE2C and does not occur with UBE2S alone, as monitored by fluorescent scanning. Uncropped gels representative of n≥3 independent experiments for **B**-**F** available in source data. Source data

With the identification of multiple Ub-binding sites, we wanted to explore their contributions to APC/C-dependent ubiquitination. In addition to UbV^CDH1^, another UbV (henceforth known as UbV^RING^) was previously developed to bind to the APC11 RING exosite and was used to trap structures of the APC/C with UBE2C and UBE2S (ref. ^23^). To specifically examine how these Ub-binding sites synergize to promote ubiquitination, we used these two UbVs to specifically disrupt these Ub-dependent interactions (Fig. 5a). First, we performed single-encounter experiments to specifically interrogate the different effects of the two UbVs in either substrate priming (the addition of the first Ub) or processive polyubiquitination. APC/C, CDH1 and a fluorescently labeled substrate are preincubated separately from a mixture of E1, E2, MgATP, Ub and a large excess of nonfluorescent substrate (Fig. 5b). Therefore, only the substrate that is preincubated with APC/C is modified during a single binding event. As expected, the UbV^RING^ reduced the number of Ubs added to the substrate during processive ubiquitination but did not disrupt substrate priming (Fig. 5c,d and Extended Data Fig. 7a). Surprisingly, the UbV^CDH1^ inhibited both reactions even though the fluorescent substrate is preincubated with APC/C^CDH1^. We then performed substrate-independent experiments to determine if the observed inhibition is specific to substrate ubiquitination or another step in the Ub cascade. Both the APC/C-dependent hydrolysis of an oxyester version of UBE2C~Ub and di-Ub synthesis by APC/C–UBE2S were unimpeded by the UbV^CDH1^ (Extended Data Fig. 7b,c). Therefore, we tested the ubiquitination of several substrates in multiple turnover experiments and found that all substrates were inhibited by UbV^CDH1^ (Extended Data Fig. 7d). To specifically test the degron dependence of these effects, we purified degron variants of Ub–Securin. Interestingly, the addition of both UbVs inhibits Ub–Securin ubiquitination when both degrons were mutated, indicating that the UbVs are preventing substrate recruitment through the added Ub (Fig. 5e and Extended Data Fig. 7e). However, UbV^CDH1^ inhibited Ub–Securin KEN^Mut^/D^Mut^ ubiquitination more than the UbV^RING^ when UBE2C was used as the E2, suggesting that the UbV^CDH1^ has additional inhibitory mechanisms.

Our experiments suggested that UbV^CDH1^ could also serve as an acceptor, receiving Ub from UBE2C instead of UBE2S. To support our hypothesis, a fluorescently labeled version of UbV^CDH1^ (*UbV^CDH1^) was subjected to ubiquitination by APC/C–UBE2C. Indeed, *UbV^CDH1^ could be modified in both a CDH1- and APC2 WHB-dependent manner (Fig. 5f). These results reveal that the CDH1 and RING Ub-binding sites promote substrate binding and improve the processivity of the APC/C. Additionally, the coactivator-bound Ub can also serve as an acceptor for Ub chain formation by UBE2C, but not UBE2S (Extended Data Fig. 7f).

Given that these interactions with Ub are probably very weak on their own, we turned to an innovative technique called mechanically transduced immunosorbent assay (METRIS) to examine the role of Ub-binding sites in providing additional affinity for the substrate^51^. METRIS is a single-particle method that can provide relative, but quantitative, assessments of binding with high statistical power. Biotinylated APC/C^CDH1^ was used to coat magnetic beads, then added to a functionalized surface of either CycB^N^ or Ub–CycB^N^, and subjected to a rotating magnetic field. The distance the bead rolls is indicative of the strength of the protein–protein interaction and is noted as the rolling parameter (RP) (Fig. 5g). Ub–CycB^N^ displayed a significant increase in its RP (0.21 ± 0.007) compared to CycB^N^ (0.15 ± 0.004), supporting the idea that Ub enhances substrate binding (Fig. 5h). Additionally, when the UbVs were added to competitively remove this noted increase, only UbV^CDH1^ substantially reduced the RP to 0.19 ± 0.007, which is still higher than the RP of APC/C^CDH1^ to CycB^N^ (Fig. 5h). To focus on the role of CDH1 in promoting the binding between APC/C and Ub, we performed experiments in the presence or absence of CDH1 and only used Ub on the functionalized surface. As expected, the addition of CDH1 to the APC/C enhanced the RP between the APC/C and Ub from 0.14 ± 0.004 to 0.17 ± 0.006 (Fig. 5i). Furthermore, the addition of both UbVs completely eliminated this improvement in the RPs to background levels (0.13 ± 0.003), suggesting that both the APC11 RING and CDH1 contribute to Ub binding. Combined, these findings suggest that multimodal interactions between substrate-linked Ubs and the coactivators, as well as the APC/C itself, work together to stabilize the emerging Ub chain and facilitate the processivity and efficiency with which the APC/C modifies its substrates (Fig. 5j).

## Discussion

To interpret molecular mechanisms, data from structural biology approaches, for example, X-ray crystallography or cryo-EM, are combined with information from biochemical or biophysical assays. Structural studies deliver static snapshots of chemically or thermodynamically stabilized machines. Biochemical assays can only monitor a restricted set of atomic arrangements within the machine at the same time. E3 Ub ligases are under intense regulation through transiently binding cofactors and perform many tasks, including recruiting different substrates, interacting with multiple E2s and forming different types of Ub chains. Therefore, trying to connect biochemical assays with structural studies to understand processive reactions remains challenging. Furthermore, understanding the driving forces and resolving key intermediate states of an evolving, Ub-modified substrate remains largely elusive. To combat this problem, we performed TR-EM experiments of the APC/C actively polyubiquitinating a substrate with its E2s.

Instead of chemically trapping the APC/C in a distinct polyubiquitination architecture, we examined APC/C^CDH1^ modifying Ub-CycB^N^ over a time course through manual plunge freezing. Using advanced deep-learning approaches for analyzing structural heterogeneity, we were able to generate a functional conformational landscape, revealing a sculpted energy landscape without high-energy populated intermediates, suggesting the absence of new thermodynamic barriers for APC/C-mediated polyubiquitination with either UBE2C or both E2s. These landscapes are stochastic with few minima or barriers. Instead, we hypothesize that thermal noise is the primary driving force and changes in the substrate, Ub and E2 interactions result in subtle allosteric changes to the APC/C scaffold that are difficult to distinctly classify. In short, there is one mode of action of the APC/C that can mediate several tasks, and subtle changes in the immediate environment, such as the ubiquitination state of the substrate or E2 bindings, modulate its function. This type of landscape can also be readily tunable by additional regulators. Since the APC/C is tightly regulated by multiple kinases, phosphatases and inhibitors, each component may only slightly alter the conformational landscape or result in different conformational pathways during different stages of the cell cycle^18,52^. While there are methodological limitations inherent to any structural method, for example, the air–water interface in cryo-EM, we reproduced several known states on the basis of structures that have been prepared in different ways from different groups, providing support for our workflow. Future work performing this type of analysis on analogous E3s can reveal how different binding partners and post-translational modifications, such as cullin neddylation, stabilize their active states^40,53^.

It was previously shown by single-molecule studies of APC/C-dependent ubiquitination that substrate-linked Ubs either help drive further catalysis through processive affinity amplification or track the Ub chain^7,54^. Our analysis revealed one previously discovered Ub-binding site on APC11 and two potential Ub-binding sites on the coactivator and UBE2C HTH^23^. We speculate that these potential Ub-binding sites could be used to enhance the processivity of polyubiquitination by increasing the substrate binding affinity for the APC/C, as suggested by our METRIS data. However, these Ub-binding sites could have additional roles during polyubiquitination. For example, Ub binding to the UBE2C^HTH^ might also allosterically modulate UBE2C activity. The Ub–coactivator interaction could similarly have multiple implications. First, because UbV^CDH1^ can serve as an acceptor, the coactivator could be presenting the Ub in a particular orientation for short chain elongation by UBE2C. Alternatively, the Ub-binding sites might be positioned in a manner to allow the substrate lysine to be modified by UBE2C instead of the Ub. This hypothesis is potentially interesting as the Ub-binding site on the coactivator is near the substrate KEN-box binding site. Since the APC/C coactivators are a key point of regulation by the cell and facilitate substrate recruitment to the APC/C, these additional Ub-binding interactions are probably subject to a series of small changes in the reaction environment and may facilitate the variety of polyubiquitination outcomes of APC/C-dependent reactions, for example, cell cycle progression. Taken together, Ub-binding sites on the substrate receptor may be a recurring theme across E3 ligases as it may help control the extent of polyubiquitination.

Several known interactions were not present in our current processing, suggesting there are probably many additional insights present in this dataset. For example, Ub binding to the APC2 WHB was not observed in our refinements^47^. Additionally, while we observed UBE2C bound to the APC/C in both datasets, the UBE2S core was never observed even though the UBE2S^CTP^ was clearly visible. The UBE2S core binds very weakly to APC2, but the UBE2S^CTP^ binds tightly to the APC2–APC4 groove^23,27,42^. Nonetheless, both E2s were bound in the APC/C^CDH1^–UBE2C–UBE2S polyubiquitination dataset. As substrate priming is typically regarded as the slow step, while chain elongation is relatively quicker, our work suggests that intrinsic differences in the reaction and/or binding kinetics of the E2s is a limiting factor for this type of approach for different E2–E3 pairs^10,12,55^.

In conclusion, we examined APC/C-mediated substrate polyubiquitination through an innovative approach, revealing the conformational landscape for substrate polyubiquitination by the APC/C. The APC/C is regulated throughout the cell cycle, and a sculpted landscape is perhaps necessary to finely tune its activity in different contexts. Furthermore, the processivity of polyubiquitination does not come directly through nucleotide binding and/or hydrolysis but rather the Ub binding to the E3. It is well established that E3 ligases are modulated by post-translational modifications and protein–protein interactions. Therefore, as we revealed several fundamental insights into APC/C-mediated polyubiquitination in relatively few experiments, we expect this method will be useful for the future studies of other E3s.

## Methods

### Protein purification

Recombinant APC/C was expressed in High Five insect cells (Thermo Scientific) as previously described^56^. APC/C with a Twin-Strep(II)-tag on the APC4 C-terminus was purified via affinity chromatography, ion-exchange chromatography and size exclusion chromatography (SEC). CDH1, UBE2C, UBE2S, UBA1, Ub, methylated Ub, Ub–CycB, Ub–Securin, Securin and CycB were purified as described^42^. For enzyme assays, CDH1 and CDC20 were expressed in baculovirus-infected insect cells with an N-terminal Myc(3x)-Hexahistidine tag and Flag(3x) tag, respectively. CDH1 and CDC20 were then subjected to Ni-NTA or anti-Flag M2 resin, respectively, and the N-terminal tags were removed by HRV 3C protease. The proteins were further purified by ion-exchange chromatography and SEC. For phage display, both CDH1 and CDC20 were purified using an uncleavable Myc(3x)-Hexahistidine tag. GST–UbV^CDH1^ and GST–Ub for co-pulldown experiments were purified using GST-affinity chromatography and SEC. Fluorescent UbV^CDH1^ was conjugated to a fluorescent LPETGG peptide via a sortase-dependent reaction and purified by buffer exchange, Ni-NTA resin and gel filtration to remove sortase and unconjugated peptide. UbV^CDH1^–Hsl1–degron fusions were designed to contain either the Hsl1 D-box (HHHHHHENLYFQSGGGMQIFVKTRKGTSITLEVEPSDTIENVK

AKIQDKEGIPPDQQRLRFAGKQLEDGRTLSDYNIHNASSLYLRSPLRALAGGSGSGSSSYLEEQKPKRAALSDITNSFNKMN) or the Hsl1 KEN-box (HHHHHHENLYFQSGGGRLRISGVSTNKENEGPEYPTKIGSGSGSGSMQIFVKTRKGTSITLEVEPSDTIENVKAKIQDKEGIPPDQQRLRFAGKQLEDGRTLSDYNIHNASSLYLRSPLRALAG). Both UbV^CDH1^–Hsl1 D-box and UbV^CDH1^–Hsl1 KEN-box were purified using nickel-affinity chromatography and SEC.

GST–TEV–AviTag–Cyclin B (1–88)–Cys–6xHis or GST–TEV–AviTag–Ub–Cyclin B (1–88)–Cys-–6xHis constructs were generated as the substrate to immobilize for TIRF microscopy or METRIS. Once purified, the substrate was biotinylated and fluorescently labeled at the C-terminal cysteine using Alexa488–maleimide.

A UBE2C–LPETGG–6xHis construct was expressed in BL21 (DE3) RIL *Escherichia* *coli* and purified. UBE2C was conjugated to a polyethylene glycol (PEG) modified peptide (GGGG–PEG–k(N_3_)NH_2_) through a sortase-dependent reaction and repurified. The UBE2C–LPETGG–PEG–k(N_3_)NH_2_ was fluorescently labeled with JF549 through click chemistry by reacting 50 μM of UBE2C with 250 μM CuSO_4_, 1.25 mM THPTA and 2.5-fold excess functionalized JF549 dye for 1.5 h on ice. The reaction was quenched with 5 mM ethylenediaminetetraacetic acid and purified using SEC.

### Synthesis of reactive Janelia dye JF549

JF549 CO2H (Tocris, 5 mg, 0.0088 mmol) was reacted with Alkyne–PEG–2NH_2_ (2.52 mg, 0.0176 mmol) in the presence of diisopropylcarbodiimide (1.633 µl, 0.0106 mmol), *N*-hydroxysuccinimide (1.52 mg, 0.0132 mmol) and diisopropylethylamine (4.59 µl, 0.026 mmol) in 500 µl of dimethylformamide (Supplementary Fig. 1). The reaction was allowed to stir at room temperature overnight. JF549-Alkyne was purified by high-performance liquid chromatography using a linear gradient of 0–100% A:B (A: 95% H_2_O:5% CH_3_CN + 0.1% trifluoroacetic acid; B: 95% CH_3_CN:5% H_2_O + 0.1% trifluoroacetic acid). The solvent was removed by rotary evaporation and further dried under high vacuum. Yield: 4.02 mg, 0.0069 mmol, 78.8%. Nuclear magnetic resonance (NMR) spectra were recorded on a 400 MHz spectrometer. NMR (400 MHz, CDCl_3_): *δ* 2.40 (t, ^4^*J* = 2.0 Hz, 1H, alkynyl CH), 2.54 (qt, ^3^*J* = 7.6 Hz, 4H, azetidinyl b-CH_2_), 3.60–3.66 (m, 8H, -OCH_2_CH_2_OCH_2_CH_2_NH-), 4.12 (d, ^4^*J* = 2.4 Hz, 2H, propargylic CH_2_), 4.22 (t, ^3^*J* = 7.6 Hz, 8H, azetidinyl a-CH_2_), 6.28 (d, ^4^*J* = 2.0 Hz, 2H), 6.34 (dd, ^3^*J*_*1*_ = 9.2 Hz, ^*4*^*J*_*2*_ = 2.0 Hz, 2H), 6.95 (d, ^3^*J* = 9.2 Hz, 2H), 7.64 (br t, ^3^*J* = 4.8 Hz, 1H, CONH-), 7.72 (s, 1H), 8.00 (d, ^3^*J* = 8.4 Hz, 1H), 8.24 (d, ^3^*J* = 8.4 Hz, 1H). Exact mass 580.24421 *m*/*z*. Observed mass 580.24484 *m*/*z*.

### Cryo-EM sample preparation

Time-resolved EM reaction components were combined in two mixes at the following concentrations: 0.7 µM APC/C, 0.7 µM CDH1, 1.25 µM Ub–CycB^N^* (mix 1) and 2.5 µM UBE2C, ± 2.5 µM UBE2S, 0.2 µM E1, 16 µM Ub and 5 mM Mg-ATP (mix 2). Mixes 1 and 2 were incubated for 5 min at room temperature, then combined to start the reaction. Samples were taken at 0.5 s, 1.5 min, 5 min and 15 min, applied to a quantifoil (1.2/1.3) 400 Cu mesh grid, blotted using a Leica plunge freezer and plunge frozen in liquid ethane. Samples were taken for SDS–PAGE at the time of plunge freezing and visualized using a Typhoon fluorescent scanner.

APC/C–CDH1–UbV^CDH1^–Hsl1 D-box complex was prepared by incubating 0.7 µM APC/C with 1.5 µM CDH1, 90 µM UbV^CDH1^–Hsl1 D-box and 0.005% fluorinated octyl maltoside. The mixture was applied to a quantifoil (1.2/1.3) 200 Cu mesh grid, blotted using a Leica plunge freezer for 2 s and plunge frozen in liquid ethane.

### Cryo-EM data acquisition

The grids were imaged using a Titan Krios (Max-Planck Institute for Multidisciplinary Science) on a K2 detector. Images were collected at a nominal magnification of 180,000× and a pixel size of 0.82 Å per pixel. Images contained 39 frames with a total dose of 42 e^−^ Å^−^^2^. A total of 25,380 micrographs were collected for the APC/C–CDH1–UBE2C dataset and 25,900 micrographs for the APC/C–CDH1–UBE2C–UBE2S dataset (Extended Data Fig. 1b).

EM samples containing APC/C–CDH1–UbV^CDH1^–Hsl1 D-box were imaged using a Titan Krios G4 (Vienna) with a cold field emission gun and a post-column Selectris energy filter (ThermoFisher) with a 10 V slit, width and a Falcon 4i direct electron detector (ThermoFisher). Images were collected at a magnification of 180,000× and a pixel size of 0.951 Å per pixel, with a cumulative total dose of 40 e^−^ Å^−^^2^.

### Image processing

Images for both datasets were motion-corrected using MotionCorr2 in Relion^57,58^. Contrast transfer function (CTF) correction was done in CryoSPARC using CTFFIND4 (refs. ^59–61^). 2D classification was carried out in CryoSPARC in three iterative rounds to filter out contamination and bad particles. The particles in the best classes were used to generate an ab initio model, with 661,289 particles in the APC/C–CDH1–UBE2C and 774,933 particles in the APC/C–CDH1–UBE2C–UBE2S dataset. This initial model was then used as a reference for 3D refinement in CryoSPARC, generating a 4.0 Å model for the APC/C–CDH1–UBE2C dataset and 3.5 Å model for the APC/C^CDH1^–UBE2C–UBE2S dataset (Fig. 1d and Extended Data Fig. 1f). A local resolution map for both final models was generated using the local resolution estimation tool in CryoSPARC (Extended Data Fig. 1d).

Images for the APC/C–CDH1–UbV^CDH1^–Hsl1 D-box dataset were preprocessed (patch motion correction and CTF estimation) using CryoSPARC Live. 2D classification was carried out in CryoSPARC in 30 iterative rounds to filter out contamination and bad particles. 3D classification was then used to select for particles containing both CDH1 and UbV^CDH1^–Hsl1 D-box density. Particles in the best class were 3D refined in CryoSPARC, generating a model at 3.88 Å (Fig. 4g).

### Model building

Cryo-EM density maps generated using 3D homogeneous refinement were sharpened using deepEMhancer and fit with existing models of APC/C using flexible fitting tools^62^. Initial atomic coordinates for both the APC/C–CDH1–UBE2C and APC/C–CDH1–UBE2C–UBE2S–Ub–CycB^N^ models were generated using the cryo-EM structure of APC/C–CDH1 (Protein Data Bank (PDB) 7qe7). Models were fitted using the fit-in-map rigid-body fitting tool in UCSF ChimeraX and refined using Isolde^63,64^. Atomic coordinates for UbV^CDH1^–Hsl1 D-box were generated using ColabFold structure prediction^65^. The cryo-EM density map for APC/C–CDH1–UbV^CDH1^–Hsl1 D-box was fitted using rigid body fitting using PDB 5a31 to fit the backbone of the APC/C and CDH1 and the ColabFold-generated structure of UbV^CDH1^–Hsl1 D-box.

### CryoDRGN heterogeneity analysis

For cryoDRGN analysis, particles from each dataset were downsampled in CryoSPARC to a 200-pixel box size (2.35 Å per pixel) and used together with CTF parameters and poses from the 3D refinement to train a cryoDRGN eight-dimensional latent variable model for 60 epochs. The encoder and decoder architectures consisted of three hidden layers with 1,024 nodes. The model was analyzed using the cryoDRGN analysis tools described in Zhong et al.^3^, to visualize the latent space and generate representative volumes and trajectories. *k*-Means clustering with *k* = 500 was used to analyze the latent embeddings of the particles, with volumes generated at the cluster centers using the decoder network to sample the heterogeneity. The latent embedding of the particle distribution was visualized using data dimensionality reduction techniques such as PCA and UMAP (Extended Data Fig. 2b). The cryoDRGN ‘analyze_landscape’ tool was used to carry out PCA on the volumes generated using *k*-means (*k* = 500) clustering (Extended Data Fig. 3a). The 500 volumes generated by the *k*-means clustering analysis were assigned manually to one of four major states (‘CRL down’, ‘CRL up’, ‘CRL unresolved’ and ‘Partial APC7). Particles from each of these four major states were extracted and 3D refinements were generated using homogeneous refinement in CryoSPARC, resulting in maps at resolutions of 4.8 Å, 4.1 Å, 4.2 Å and 4.3 Å, respectively (Fig. 2b and Extended Data Fig. 4a). The minor states ‘CRL down, CDH1 bound’, ‘CRL down, CDH1 unbound’, ‘CRL up, partial’, ‘CRL up, no UBE2C’ and ‘CRL up, UBE2C bound’ were assigned by visual inspection of the major states.

### Coverslip preparation

Glass coverslips (Corning) were cleaned, functionalized through silanization and PEGylation, and assembled into flow chambers for TIRF microscopy experiments. All buffers were 0.22 μm sterile filtered. Coverslips were sonicated for 30 min in 1 M KOH, 30 min in 200-proof EtOH, then 20 min in deionized (DI) water. Coverslips were silanized by sonicating for 5 min in MeOH, rocked for 12 min in a solution of 93.5% MeOH, 5% acetic acid and 1.5% (3-aminopropyl) triethoxysilane, sonicated for 1 min, rocked in solution for another 12 min, then sonicated for 60 min in DI water. Pairs of coverslips were sandwiched together with a solution of 100 mM NaHCO_3_, 500 mM K_2_SO_4_, 18% succinimidyl valerate–PEG and 0.4% biotin–PEG, then incubated in the dark overnight in humid conditions.

Glass slides (Fisher Scientific) were modified with diamond drill bits to have three pairs of holes on opposite sides of the slide. Thin strips of double-sided tape were laid across the width of the slide on the outside of the holes and between each pair. Functionalized coverslips were washed with DI water, dried and placed on top of the taped slide, then sealed with coverslip sealant to create flow chambers.

Once assembled, flow chambers were incubated with 100 mg ml^−1^ bovine serum albumin (BSA) in assay buffer (20 mM HEPES pH 8 and 200 mM NaCl, 0.22 μm filtered) for 30 min, then a 0.5 mg ml^−1^ neutravidin and 100 mg ml^−1^ BSA mixture for 5 min, then a 50 nM Cyclin B–Alexa488 and 20 mg ml^−1^ BSA mixture for 2 min. The chambers were washed after each incubation step.

### TIRF microscopy

TIRF microscopy was performed on an IX81 Olympus microscope equipped with a 100× TIRF objective (UPLAPO100XOHR, numerical aperture 1.5, Olympus), four solid-state OBIS lasers (Coherent OBIS: 405 nm, 488 nm, 561 nm and 647 nm; controlled via CoherentConnection software), and two scientific complementary metal–oxide–semiconductor cameras for dual-color imaging. The microscope was controlled using MicroManager software^66^.

An initial image of the immobilized substrate within flow chambers was taken with the 488 nm laser at 4 mW power to verify substrate attachment and determine the region of interest for subsequent analysis. Experiment movies were collected using the 561 nm laser at 2 mW power with 200 ms exposure over a 10 min period after adding reaction components to the flow chamber.

Experimental reactions were made by premixing a phosphomimetic version of APC/C^36,67^ and CDH1 (mix 1), and premixing BSA, UBE2C–JF549 and Ub (mix 2). Final reaction mixtures contained 30 nM pE APC, 100 nM CDH1, 20 mg ml^−1^ BSA, 200 nM UBE2C–JF549, 5 μM Ub, 100 nM UBA1, 0.8% d-glucose, 5.6 mg ml^−1^ glucose oxidase, 0.34 mg ml^−1^ catalase, 2 mM TROLOX and a titration of UBE2S CTP. These mixtures were combined with 5 mM ATP-MgCl_2_ and added to flow chambers before data acquisition. To validate that our system, ubiquitination of 50 nM biotin–CycB^N^–A488 was monitored over a short time course using SDS–PAGE (Extended Data Fig. 5c).

### TIRF microscopy data analysis

For each condition, a set of *N* = 3 experiments were carried out. ImageJ/FIJI was used to extract a standardized region of interest from experimental movies. In MATLAB 2023a, images were background-subtracted, and single molecules were isolated using an algorithm based on á trous wavelet decomposition^68^. Long-lasting events were linked across frames using a well-established algorithm^69^. Our analysis utilized the MATLAB Curve Fitting Toolbox, Image Processing Toolbox, Microscopy Image Browser, and the Statistics and Machine Learning Toolbox. For each condition, outliers were removed using the ROUT method in GraphPad Prism (Q = 1%) (ref. ^70^). Detected binding events for each experiment were normalized relative to the condition without APC/C.

**Table 1:** Cryo-EM data collection, refinement and validation statistics

	#1 APC/C–CDH1–UBE2C	#2 APC/C–CDH1–UBE2C–UBE2S
	(EMD-41140), (PDB 8TAR)	(EMD-41142), (PDB 8TAU)
**Data collection and processing**		
Magnification	180,000×	180,000×
Voltage (kV)	300	300
Electron exposure (e^−^ Å^−^^2^)	42	42
Defocus range (μm)	−0.7 to −3.5	−0.7 to −3.5
Pixel size (Å)	0.82	0.82
Symmetry imposed	C1	C1
Initial particle images (no.)	2,001,820	1,894,448
Final particle images (no.)	661,289	774,933
Map resolution (Å)	4.0 Å	3.5 Å
FSC threshold	0.143	0.143
Map resolution range (Å)	3–10 Å	3–10 Å
**Refinement**		
Initial model used (PDB code)	7QE7	7QE7
Model resolution (Å)	4.0 Å	3.5 Å
FSC threshold	0.143	0.143
Model resolution range (Å)	3–10 Å	3–10 Å
Map sharpening *B* factor (Å^2^)	−188.3	−159.6
Model composition		
Non-hydrogen atoms	35,965	36,038
Protein residues	8,989	9,007
R. m. s. deviations		
Bond lengths (Å)	0.006	0.007
Bond angles (°)	0.766	0.792
**Validation**		
MolProbity score	1.70	1.81
Clashscore	6.84	9.17
Ramachandran plot		
Favored (%)	98.19%	97.89%
Allowed (%)	1.81%	2.12%
Disallowed (%)	0.00%	0.00%

### Local heterogeneity analysis

Particles assigned the ‘CRL up’ major state were extracted and imported into CryoSPARC for further analysis^61^. Particles were classified into separate 3D classes using the ‘3D Classification Beta’ tool for local classification using a mask including only the catalytic core of the APC/C (CDH1, APC10, UBE2C, APC11 and the APC2^CRL^ domains). Particles from each class were further refined using 3D homogeneous refinement and fitted using rigid body fitting in UCSF ChimeraX.

### Ubiquitination assays

In general, fluorescent substrate ubiquitination assays were performed at room temperature, quenched by SDS-containing sample buffer, separated by SDS–PAGE, and visualized using a Typhoon Fluorescence scanner. Ubiquitination assays testing inhibition of substrate polyubiquitination were carried out by mixing 70 nM APC/C, 0.5 µM CDH1, 5 mM ATP/MgCl_2_, 1 µM E1, 1 µM UBE2C, 0.5 µM Ub–CycB^N^*, ±30 µM UbV^CDH1^, ±Hsl1 D-box, ±Hsl1 KEN-box, ±UbV^CDH1^–Hsl1 D-box or ±UbV^CDH1^–Hsl1 KEN-box. A total of 125 µM Ub was added to start the reaction and quenched after 15 min. Single-encounter assay conditions were carried out by first incubating two separate mixes for 5 min: mix 1 containing APC/C–CDH1, either CycB^N^* or a version of CycB^N^* containing a single lysine and ±30 µM UbV^RING^ or UbV^CDH1^, and mix 2 containing 0.1 µM E1, 5 µM UBE2C, 5 mM ATP/MgCl_2_, excess unlabeled Hsl1 D-box at 48 µM, and either Ub or methyl-Ub at 125 µM. The two mixtures were combined and then quenched after 3 min. Bands containing either total modified substrate or substrate modified with >4 Ubs were quantified and normalized relative to the wild-type levels of ubiquitination. Values were plotted using GraphPad Prism. Substrate ubiquitination reactions testing Ub–Securin degron mutants were carried out by combining 70 nM APC/C, 0.5 µM CDH1, 5 mM ATP/MgCl_2_, 1 µM E1, 1 µM UBE2C, 0.5 µM Ub–Securin*, ±30 µM UbV^CDH1^, ±30 µM UbV^RING^, and 125 µM Ub added to start the reaction, then quenched after 15 min. Ubiquitination assays testing different E2 and substrate combinations were carried out in a similar manner with 70 nM APC/C, 0.5 µM CDC20, 5 mM ATP/MgCl_2_, 1 µM E1, 1 µM E2 (UBE2C, UBCH5B and/or UBE2S), 0.5 µM substrate (CycB^N^* or Ub-CycB^N^*, Securin* or Ub-Securin*) and ±30 µM UbV^CDH1^. *UbV^CDH1^ ubiquitination reactions were carried out using 50 nM APC/C or APC/C^∆WHB^, ±0.5 µM CDH1, 7 µM *UbV^CDH1^, 2 µM UBE2C and/or UBE2S, 1 µM E1, 5 mM ATP/MgCl_2_ and 125 µm Ub, and carried out at 0, 30 and 60 min timepoints.

Intrinsic ability of APC/C to catalyze the hydrolysis of Ub from UBE2C was tested using an oxyester-linked UBE2C~Ub (where ~ denotes oxyester linkage) in the presence and absence of coactivator or UbV^CDH1^. Reaction mixtures contained 1 µM APC/C, 5 µM UBE2C~Ub and ±10 µM UbV^CDH1^ and were quenched after 0, 3 or 5 h at room temperature and visualized using SDS–PAGE and Coomassie staining. Di-Ub synthesis assays were carried out as described previously, with the addition of ±10 µM UbV^CDH1^(ref. ^23^).

### Phage display

The UbV library was based upon the same design as library 2 generated by Ernst et al.^50^. Phage display selection was carried out according to established protocols^48^. Briefly, 1 μM purified CDH1 and CDC20 were coated on 96-well MaxiSorp plates (Thermo Scientific) by incubating overnight at 4 °C. Five rounds of selection using the phage-displayed UbV library were performed against immobilized proteins. The coated wells were blocked with PBS-B (phosphate-buffered saline (PBS) supplemented with 1% BSA) and incubated at 4 °C for 1 h. The original phage display library (first round) or the enriched input from the previous round (subsequent rounds) was added to each coated well and incubated for 1 h at room temperature. Non-bound phages were removed by washing the coated wells with PBS-T (PBS supplemented with 0.05% Tween-20). Washing stringency was increased with each round. To elute the bound phage, 0.1 M HCl was added to each coated well and incubated for 5 min at room temperature. The pH was neutralized by adding 1 M Tris–HCl (pH 11), and BSA was added to eluted phage to a final concentration of 1%. Amplifying the eluted phage to generate input phage for subsequent rounds was achieved by superinfecting *E. coli* OmniMax with both eluted phage and helper phage. After the fifth round of selection, phage generated in rounds 4 and 5 were analyzed by phage ELISA to obtain crude binding data and their corresponding phagemids were subjected to Sanger sequencing to obtain UbV sequences and assess binder enrichment. The CDC20 selection produced the coactivator-binding UbV used in this study.

### APC/C substrate degradation assay using CP HeLaS3 extracts

The degradation assay using a checkpoint (CP) cell-free extract was performed essentially as described previously^45,71^. HeLaS3 cells were grown at 37 °C with 5% CO_2_ in Dulbecco’s modified Eagle medium (DMEM, Gibco) supplemented with 10% fetal bovine serum. Confluent cells were seeded in 15 cm plates at 3 million cells per plate and treated the day after with 2 mM thymidine for 24 h. Thymidine-containing medium was removed, and cells were washed with warm PBS twice and drug-free DMEM once, and released in DMEM for 4 h. Then, cells were treated with 100 ng ml^−1^ of nocodazole in DMEM for 11 h to obtain an early mitotic (prometaphase) population and collected for extract preparation. Mitotic cells (>95%) were collected by ‘shake off’, pelleted at 277*g*, washed with PBS once then resuspended in 1 ml of ice-cold PBS and pelleted in Eppendorf tubes. Cell pellets from 4 × 15 cm dishes were lysed in 600 µl of SB buffer (20 mM HEPES pH 7.5, 1.5 mM MgCl_2_, 1 mM dithiothreitol, 5 mM KCl, 2 μg ml^−1^ pepstatin, 2 μg ml^−1^ apoprotinin, 10 μg ml^−1^ leupeptin, 1 mM 4-(2 aminoethyl) benzenesulfonyl fluoride and 1 mM Na_3_VO_4_) supplemented with 20 μl of energy mix (375 mM creatine phosphate, 50 mM ATP and 50 mM MgCl_2_, pH 8.0). Cells were incubated for 30 min on ice and flipped every 5 min before being frozen in liquid nitrogen then quickly thawed at 30 °C, and the procedure was repeated one more time. DNA was sheared by passing the lysate seven times through a 20 1/2 G and twice through a 25 3/8 G. The lysate was centrifugated at 3,075*g* at 4 °C for 5 min first, and its supernatant was then centrifuged at 24,104*g* at 4 °C for 30 min. The resulting extract was aliquoted and either stored at −80 °C or used immediately. Typically, a master mix made of 50 μl of CP extracts was supplemented with 12 μl of energy mix, 12 μl of either 850 μM Ub or UbV^CDH1^ and 3 μl of 40 μM UBE2C. The master mix was diluted 1:1 in fresh SB buffer minus proteases inhibitors, and reactions were incubated at 30 °C. Aliquots were taken out at the indicated times, stopped with sample buffer, boiled and analyzed by SDS–PAGE and western blot.

### METRIS

The METRIS experiments were performed similarly to previously described methods^51^. The APC/C harbored a multipurpose tag on the C-terminus of APC4 that included a HaloTag, AviTag and Twin-Strep(II)-tag. After ion exchange chromatography described above, the APC/C was biotinylated and subjected to SEC. Ub was biotinylated on an N-terminal cysteine using biotin maleimide.

Biotinylated Ub, CycB or Ub-CycB was added to fully saturate and coat streptavidin high-capacity eight-well strips and left to bind for 2 h (Sigma-Aldrich). The wells were then washed with a blocking buffer and left for 30 min after which they were washed again. The biotinylated APC/C was added in 50-fold excess to the theoretical capacity of streptavidin-coated ferromagnetic beads (Spherotech). Biotin-APC/C (3 μM) and CDH1 (15 μM) were incubated with ferromagnetic beads for 2 hours and the solution of magnetic beads was diluted 5,000-fold when the solution of magnetic beads was inserted into the well and magnetized using a permanent neodymium magnet. The well was placed into the Helmholtz-coil like apparatus and the rotating magnetic field was actuated at a frequency of 1 Hz for all experiments. Data acquisition was achieved using a camera for visualization, video capture and subsequent analysis described previously^51^. The RP is the distance the bead travels (∆*x*) divided by the maximum theoretical displacement of a sphere, calculated using the following equation:

$${{\mathrm{RP}}}=\frac{\Delta x}{\pi D\tau \omega }$$

where *D* is the particle diameter, *ω* is the frequency of the rotating magnetic field and *τ*is the actuation time.

### Reporting summary

Further information on research design is available in the Nature Portfolio Reporting Summary linked to this article.

## Online content

Any methods, additional references, Nature Portfolio reporting summaries, source data, extended data, supplementary information, acknowledgements, peer review information; details of author contributions and competing interests; and statements of data and code availability are available at 10.1038/s41594-023-01105-5.

### Supplementary information

Supplementary Information Supplementary Fig. 1.

Reporting Summary

Supplementary Video 1 Trajectories from the PCA of the particle distribution in the latent space show volumes generated along PC1 and PC2 of the latent space and map out the CRL transitioning from the ‘CRL down’ to the ‘CRL up’ conformation as well as heterogeneity near the coactivator and active site.

Supplementary Video 2 Representative movies of UBE2C–JF549 signal across a titration of UBE2S^CTP^. All movies are taken using the UBE2C channel monitoring UBE2C–JF549 binding events in flow cells with immobilized Biotin–CycB^N^*–A488. Exposure: 200 ms. The first 300 frames of 3,000-frame movies are shown.

### Source data

Source Data Fig. 1 Unprocessed western blots and/or gels.

Source Data Fig. 3 Statistical source data.

Source Data Fig. 4 Unprocessed western blots and/or gels.

Source Data Fig. 5 Unprocessed western blots and/or gels.

Source Data Fig. 5 Statistical source data.

Source Data Extended Data Fig. 5 Statistical source data.

Source Data Extended Data Fig. 5 Unprocessed western blots and/or gels.

Source Data Extended Data Fig. 6 Unprocessed western blots and/or gels.

Source Data Extended Data Fig. 7 Unprocessed western blots and/or gels.

Source Data Extended Data Fig. 7 Statistical source data.

## Data Availability

The cryo-EM density maps of APC/C–CDH1–UBE2C–Ub–CycB^N^ and APC/C–CDH1–UBE2C–UBE2S–Ub–CycB^N^ were deposited in the Electron Microscopy Data Bank under accession numbers EMD-41140 and EMD-41142, respectively. The corresponding atomic coordinates were deposited in the RCSB Protein Data Bank under accession numbers 8TAR (APC/C–CDH1–UBE2C–Ub–CycB^N^) and 8TAU (APC/C–CDH1–UBE2C–UBE2S–Ub–CycB^N^). The raw EM data, trained cryoDRGN models and generated volumes from this study are available on Electron Microscopy Public Image Archive database (EMPIAR-11660 and EMPIAR-11661). Unprocessed images and numerical raw data are provided as source data. Additional data available upon request. Source data are provided with this paper.
