# Supplementary material for: Time-resolved cryo-EM (TR-EM) analysis of substrate polyubiquitination by the RING E3 anaphase-promoting complex/cyclosome (APC/C)
Source: Nat Struct Mol Biol. 2023 Sep 21;30(11):1663–74. doi: 10.1038/s41594-023-01105-5 (PMC10643132; doi:10.1038/s41594-023-01105-5)

nature structural & molecular biology

Article

https://doi.org/10.1038/s41594-023-01105-5

Time-resolved cryo-EM (TR-EM) analysis of substrate polyubiquitination by the RING E3 anaphase-promoting complex/cyclosome (APC/C)

In the format provided by the authors and unedited

Supplementary Fig. 1

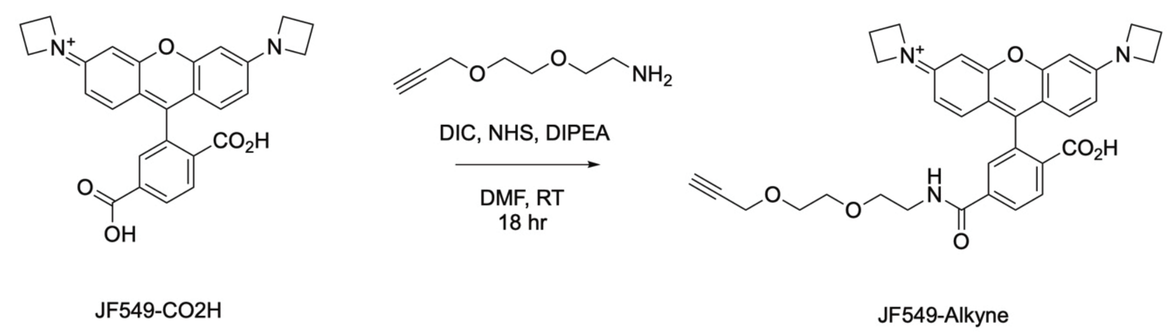

Supplement: Supplementary file 1 — Supplementary Fig. 1. [file 41594_2023_1105_MOESM1_ESM.pdf]
