# Supplementary material for: Time-resolved cryo-EM (TR-EM) analysis of substrate polyubiquitination by the RING E3 anaphase-promoting complex/cyclosome (APC/C)
Source: Nat Struct Mol Biol. 2023 Sep 21;30(11):1663–74. doi: 10.1038/s41594-023-01105-5 (PMC10643132; doi:10.1038/s41594-023-01105-5)
Supplement: Supplementary file 2 — Reporting Summary [file 41594_2023_1105_MOESM2_ESM.pdf]

nature portfolio

Corresponding author(s): Nicholas G. Brown, David Haselbach

Last updated by author(s): Aug 21, 2023

Reporting Summary

Nature Portfolio wishes to improve the reproducibility of the work that we publish. This form provides structure for consistency and transparency in reporting. For further information on Nature Portfolio policies, see our Editorial Policies and the Editorial Policy Checklist.

Statistics

For all statistical analyses, confirm that the following items are present in the figure legend, table legend, main text, or Methods section.

n/a	Confirmed	
☐	☒	The exact sample size (*n*) for each experimental group/condition, given as a discrete number and unit of measurement
☐	☒	A statement on whether measurements were taken from distinct samples or whether the same sample was measured repeatedly
☐	☒	The statistical test(s) used AND whether they are one- or two-sided<br>*Only common tests should be described solely by name; describe more complex techniques in the Methods section.*
☒	☐	A description of all covariates tested
☐	☒	A description of any assumptions or corrections, such as tests of normality and adjustment for multiple comparisons
☐	☒	A full description of the statistical parameters including central tendency (e.g. means) or other basic estimates (e.g. regression coefficient) AND variation (e.g. standard deviation) or associated estimates of uncertainty (e.g. confidence intervals)
☐	☒	For null hypothesis testing, the test statistic (e.g. *F*, *t*, *r*) with confidence intervals, effect sizes, degrees of freedom and *P* value noted<br>*Give P values as exact values whenever suitable.*
☒	☐	For Bayesian analysis, information on the choice of priors and Markov chain Monte Carlo settings
☒	☐	For hierarchical and complex designs, identification of the appropriate level for tests and full reporting of outcomes
☒	☐	Estimates of effect sizes (e.g. Cohen's *d*, Pearson's *r*), indicating how they were calculated

Our web collection on statistics for biologists contains articles on many of the points above.

Software and code

Policy information about availability of computer code

Data collection	ThermoFisher EPU 1.9 and 2.1 software was used to collect cryoEM microscopy images.<br>Microscopy images were collected using a microscope controlled by MicroManager 2.0.0-gamma1 software and OBIS lasers controlled using CoherentConnection v4.0.0.28 software.<br>All fluorescent imaging was performed on an Amersham Typhoon 5.<br>Metris experiment measurements were made using a CMOS camera.
Data analysis	Electron microscopy images were analyzed using RELION 3.0, cryosparc v4.2.1, and cryoDRGN v0.3.1.<br>Fluorescent microscopy images were analyzed using ImageJ/FIJI 2.3.0/1.53t, Matlab 2022a (packages: Curve Fitting Toolbox v3.8, Image Processing Toolbox v11.6, Microscopy Image Browser v1.302.0.0, and the Statistics and Machine Learning Toolbox v12.4), and Graphpad Prism 9.<br>Ubiquitinated product bands were quantified using ImageJ 2.0.0-rc-69/1.52p and data was plotted using Graphpad Prism 9.<br>Metris measurements were analyzed using Graphpad Prism 9.

For manuscripts utilizing custom algorithms or software that are central to the research but not yet described in published literature, software must be made available to editors and reviewers. We strongly encourage code deposition in a community repository (e.g. GitHub). See the Nature Portfolio guidelines for submitting code & software for further information.

Data

Policy information about availability of data

All manuscripts must include a data availability statement. This statement should provide the following information, where applicable:

- Accession codes, unique identifiers, or web links for publicly available datasets
- A description of any restrictions on data availability
- For clinical datasets or third party data, please ensure that the statement adheres to our policy

The cryo-EM density maps of APC/C-CDH1-UBE2C-Ubiquitin-CycBN and APC/C-CDH1-UBE2C-UBE2S-Ubiquitin-CycBN were deposited in the Electron Microscopy Data Bank under accession numbers EMD-41140 and EMD-41142, respectively. The corresponding atomic coordinates were deposited in the RCSB Protein Data Bank under accession numbers 8TAR (APC/C-CDH1-UBE2C-Ubiquitin-CycBN) and 8TAU (APC/C-CDH1-UBE2C-UBE2S-Ubiquitin-CycBN). The raw EM data, trained cryoDRGN models, and generated volumes from this study are available on Electron Microscopy Public Image Archive database (EMPIAR-11660 and EMPIAR-11661). Unprocessed images and numerical raw data are provided as source data. Additional data available upon request.

Human research participants

Policy information about studies involving human research participants and Sex and Gender in Research.

Reporting on sex and gender	N/A
Population characteristics	N/A
Recruitment	N/A
Ethics oversight	N/A

Note that full information on the approval of the study protocol must also be provided in the manuscript.

Field-specific reporting

Please select the one below that is the best fit for your research. If you are not sure, read the appropriate sections before making your selection.

☒ Life sciences ☐ Behavioural & social sciences ☐ Ecological, evolutionary & environmental sciences

For a reference copy of the document with all sections, see nature.com/documents/nr-reporting-summary-flat.pdf

Life sciences study design

All studies must disclose on these points even when the disclosure is negative.

Sample size	Sample sizes are indicated in relevant figure and methods sections.
Data exclusions	No data was excluded.
Replication	All experiments were repeated at least three times, all attempts to repeat these experiments were successful.
Randomization	Randomization was not necessary or required for the data analyzed in this study, grouped data was not included in the study.
Blinding	Blinding was not relevant to our study, grouped data was not used in the study.

Reporting for specific materials, systems and methods

We require information from authors about some types of materials, experimental systems and methods used in many studies. Here, indicate whether each material, system or method listed is relevant to your study. If you are not sure if a list item applies to your research, read the appropriate section before selecting a response.

Materials & experimental systems

n/a	Involved in the study
☐	☒ Antibodies
☐	☒ Eukaryotic cell lines
☒	☐ Palaeontology and archaeology
☒	☐ Animals and other organisms
☒	☐ Clinical data
☒	☐ Dual use research of concern

Methods

n/a	Involved in the study
☒	☐ ChIP-seq
☒	☐ Flow cytometry
☒	☐ MRI-based neuroimaging

Antibodies

Antibodies used	α-Securin 1:1,000 dilution (sc-56207), α-Vinculin 1:5,000 dilution (sc-25336), α-Geminin 1:5,000 (sc-13015), α-CyclinB 1:10,000 dilution (ab32053), α-Cyclin A 1:5,000 dilution (sc-751)
Validation	Validation of the antibodies was performed by the manufacturer. Western blotting results were consistent with Coomassie stained SDS PAGE analysis and known cell cycle degradation patterns.

Eukaryotic cell lines

Policy information about cell lines and Sex and Gender in Research

Cell line source(s)	Insect cells were from life technologies, Hi Five Cat# B85502 and Sf9 Cat#11496015.<br>HeLaS3 cells were obtained from ATCC (CCL-2.2, Manassas, Virginia)
Authentication	None of the cell lines were authenticated.
Mycoplasma contamination	All cells were negative for mycoplasma.
Commonly misidentified lines (See ICLAC register)	No commonly misidentified lines were used.
