# Supplementary figures and images for: Time-resolved cryo-EM (TR-EM) analysis of substrate polyubiquitination by the RING E3 anaphase-promoting complex/cyclosome (APC/C)
Source: Nat Struct Mol Biol. 2023 Sep 21;30(11):1663–74. doi: 10.1038/s41594-023-01105-5 (PMC10643132; doi:10.1038/s41594-023-01105-5)

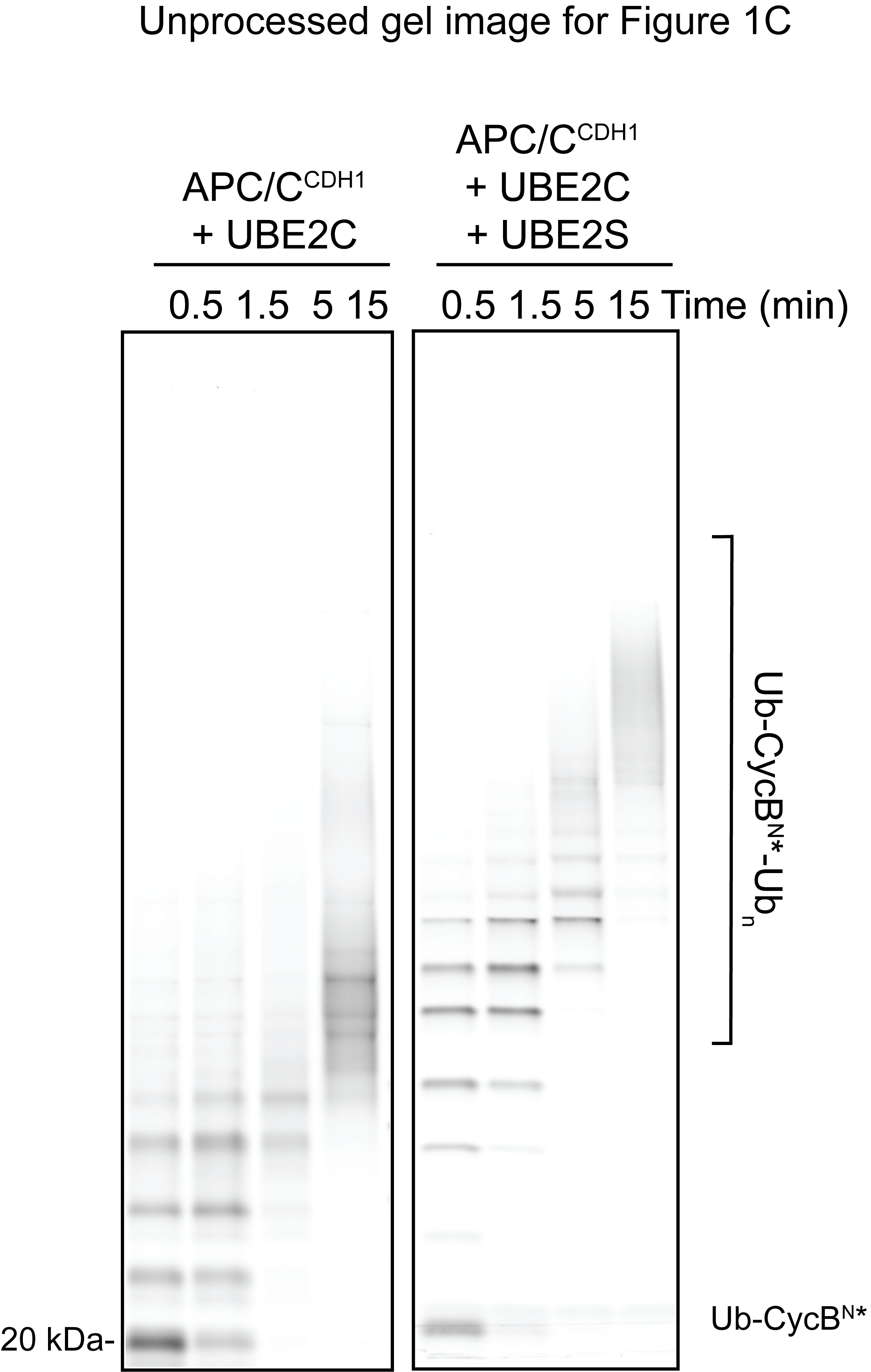

Supplement: Supplementary file 5 — Unprocessed western blots and/or gels. [file 41594_2023_1105_MOESM5_ESM.tif]

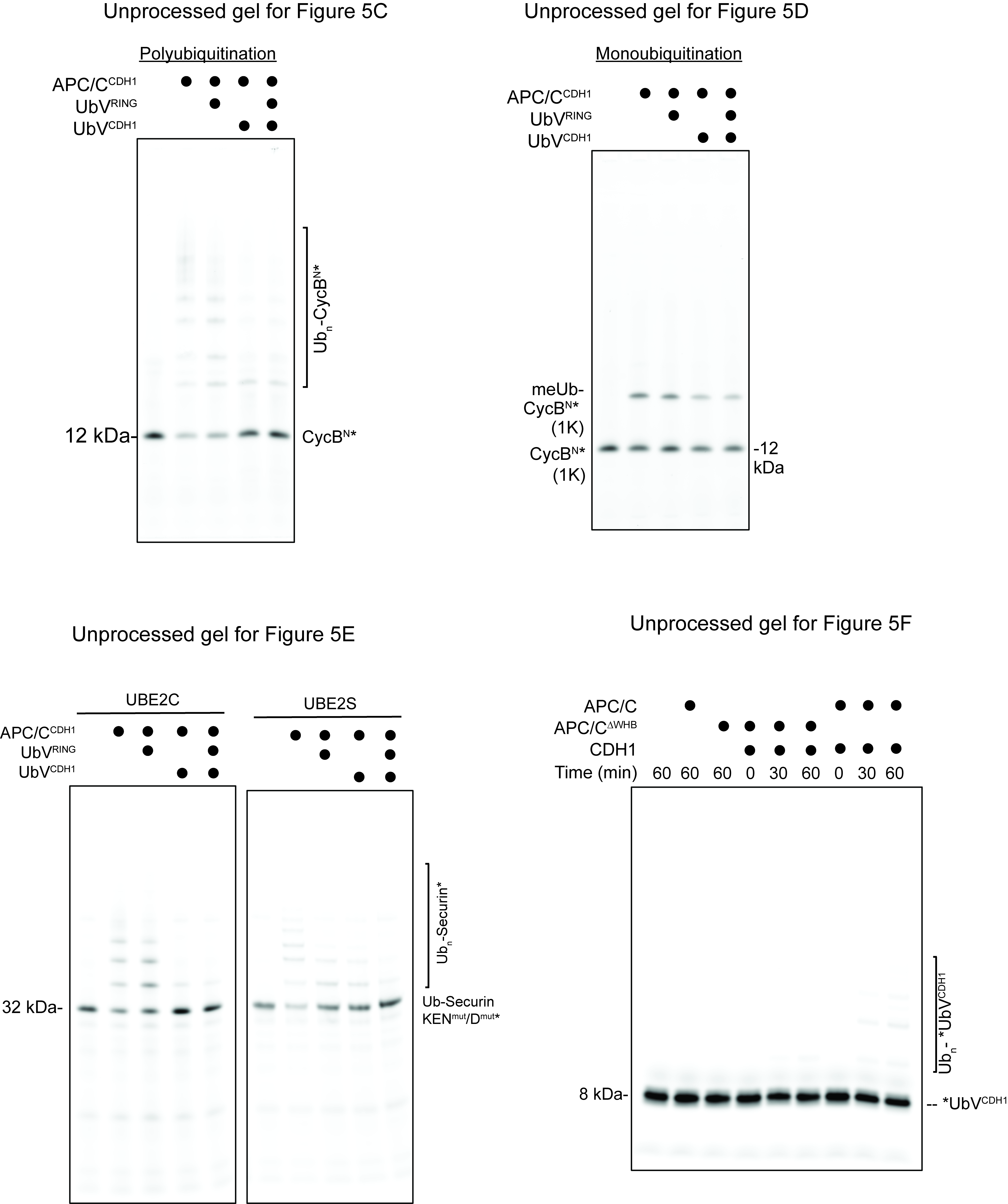

Supplement: Supplementary file 8 — Unprocessed western blots and/or gels. [file 41594_2023_1105_MOESM8_ESM.tif]

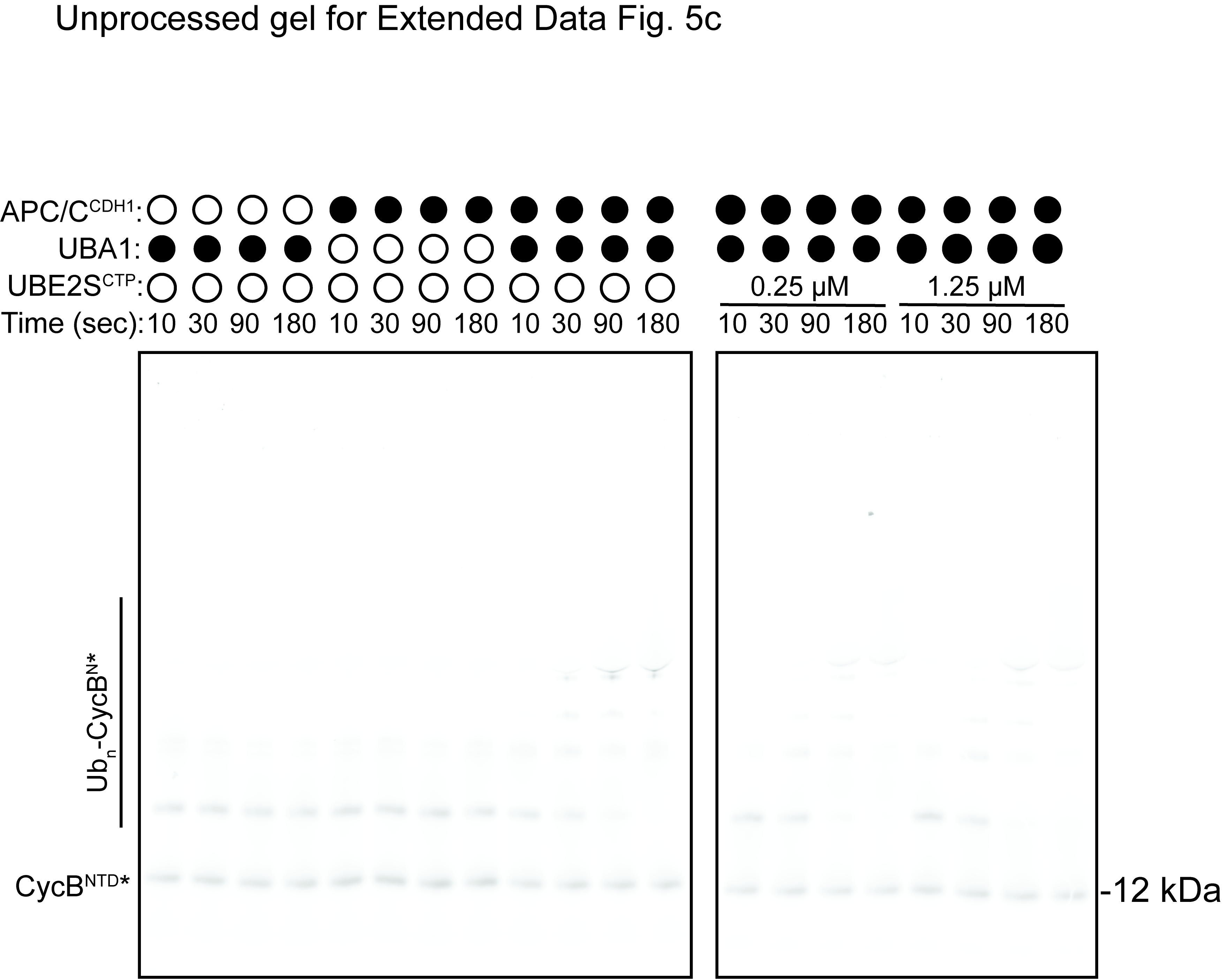

Supplement: Supplementary file 11 — Unprocessed western blots and/or gels. [file 41594_2023_1105_MOESM11_ESM.tif]

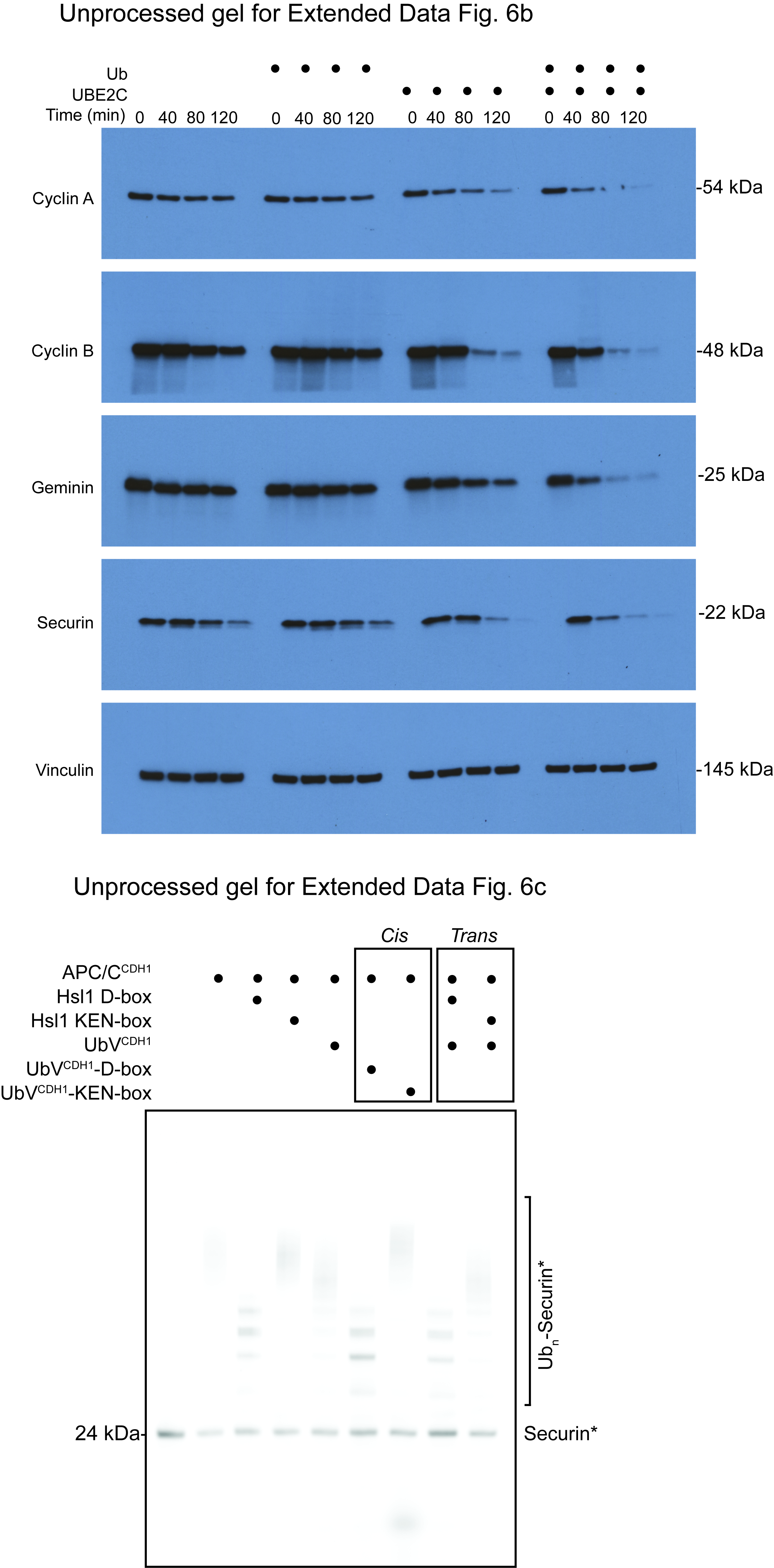

Supplement: Supplementary file 12 — Unprocessed western blots and/or gels. [file 41594_2023_1105_MOESM12_ESM.tif]
